# CTCF-mediated insulation and chromatin environment modulate *Car5b* escape from X inactivation

**DOI:** 10.1186/s12915-025-02137-7

**Published:** 2025-03-03

**Authors:** He Fang, Ana R. Tronco, Giancarlo Bonora, Truong Nguyen, Jitendra Thakur, Joel B. Berletch, Galina N. Filippova, Steven Henikoff, Jay Shendure, William S. Noble, Zhijun Duan, Christine M. Disteche, Xinxian Deng

**Affiliations:** 1 Department of Laboratory Medicine and Pathology, University of Washington, Seattle, WA 98195 USA; 2 Department of Genome Sciences, University of Washington, Seattle, WA 98195 USA; 3 Basic Sciences Division, Fred Hutchinson Cancer Research Center, Seattle, WA 98109 USA; 4 Paul G. Allen School of Computer Science and Engineering, University of Washington, Seattle, WA 98195 USA; 5 Institute for Stem Cell and Regenerative Medicine, University of Washington, Seattle, WA 98195 USA; 6 Division of Hematology, University of Washington, Seattle, WA 98195 USA; 7 Department of Medicine, University of Washington, Seattle, WA 98195 USA

**Keywords:** CTCF, Chromatin looping, Insulation, X-chromosome inactivation, Escape from X-chromosome inactivation

## Abstract

**Background:**

Genes that escape X-chromosome inactivation (XCI) in female somatic cells vary in number and levels of escape among mammalian species and tissues, potentially contributing to species- and tissue-specific sex differences. CTCF, a master chromatin conformation regulator, is enriched at escape regions and may play an important role in regulating escape, but the molecular mechanisms remain elusive.

**Results:**

CTCF binding profiles and epigenetic features were systematically examined at escape genes (escapees) using mouse allelic systems with skewed XCI to distinguish the inactive X (Xi) and active X (Xa) chromosomes. We found that six constitutive and two facultative escapees are located inside 30-800 kb domains marked by convergent arrays of CTCF binding sites, consistent with the formation of chromatin loops. Facultative escapees show clear differences in CTCF binding depending on their XCI status in specific cell types/tissues. In addition, sets of strong and in some cases divergent CTCF binding sites located at the boundary between an escapee and its adjacent neighbors subject to XCI would also help insulate domains. Indeed, deletion but not inversion of a CTCF binding site at the boundary between the facultative escapee *Car5b* and its silent neighbor *Siah1b* results in a dramatic reduction of *Car5b* escape. This is associated with reduced CTCF and cohesin binding, which indicates loss of looping and insulation and is supported by 3C combined with Hi-C analysis. In addition, enrichment in the repressive mark H3K27me3 invades the *Car5b* domain in deleted cells, consistent with loss of expression from the Xi*.* In contrast, cells with an inversion of the CTCF binding site retain CTCF and cohesin binding, as well as looping, in line with persistence of escape. Interestingly, the levels of escape increase in cells with deletion of either *Dxz4*, which disrupts the Xi-specific compact 3D structure, or *Firre*, which results in lower H3K27me3 enrichment on the Xi, indicating that the structural and epigenetic features of the Xi constrain escape from XCI in wild type conditions.

**Conclusions:**

Taken together, our findings support the idea that escape from XCI in female somatic cells is modulated by both the topological insulation of domains via CTCF binding and the surrounding heterochromatin environment.

**Supplementary Information:**

The online version contains supplementary material available at 10.1186/s12915-025-02137-7.

## Background

X-chromosome inactivation (XCI) silences one of the two X chromosomes in mammalian females to equalize X-linked gene expression between males (XY) and females (XX) [1]. XCI is a chromosome-wide epigenetic process orchestrated by a long non-coding RNA *Xist* (X-inactive specific transcript) during early female embryogenesis [2–6]. *Xist* RNA cis-coats the future inactive X chromosome (Xi) and recruits or removes specific proteins and chromatin modifications to silence genes. As a result, the Xi acquires a specific pattern of epigenetic hallmarks by depletion of active histone modifications and enrichment in repressive histone modifications. Xi-specific features also include enrichment of the histone variant macroH2A, hyper-methylation of CpG islands, and late DNA replication. In addition, the Xi becomes highly condensed and is reshaped into a heterochromatic bipartite structure containing two compact superdomains of long-range chromatin interactions separated by a boundary at the conserved macrosatellite repeat *Dxz4*. The multi-layer epigenetic regulation of the Xi ensures that its silent state is stably maintained in somatic cells. However, some genes escape XCI, including about 15–30% of human and 3–7% of mouse X-linked genes, resulting in higher expression levels in females compared to males, which potentially contributes to phenotypic sex differences in development and health [7–13]. The number and levels of genes that escape XCI vary among species, tissues, and cell types [14–19]. Some genes (“constitutive escapees”) escape XCI in a ubiquitous manner, while others (“facultative escapees”) only escape in certain cell types and tissues, resulting in specific sex differences. Most escapees have lower expression from the Xi compared to that from the active X chromosome (Xa), suggesting partial repression of these genes due to proximity to heterochromatin.

The molecular mechanisms by which escapees partly evade the multiple layers of heterochromatin control inherent to XCI and the factors that regulate expression levels from the Xi in specific cell types, tissues, and species are not well understood. Identifying these mechanisms would help define the roles of escapees in sex differences. While chromatin changes have been correlated with escape [11, 20, 21], it is unclear how these changes are regulated. Emerging evidence suggests that an important master regulator of chromatin structure, CTCF (CCCTC-binding factor), may play an important role in regulating chromatin insulation and escape from XCI. We previously reported that CTCF binds to the 5’ end of two mouse constitutive escapees, *Kdm5c* and *Eif2s3x,* where it might function as a boundary to insulate these genes from neighbors subject to XCI [22]. In support of this model, CTCF profiles we obtained by allele-specific ChIP-seq in two systems with skewed XCI, a fibroblast cell line (Patski) and adult tissues from F1 hybrid mice, have clearly shown that CTCF binding is reduced on the Xi versus the Xa, except at escape regions [16]. Interestingly, *Car5b* that escapes XCI in Patski cells but not in mouse brain is flanked by CTCF binding sites in Patski cells only, supporting the role of CTCF in regulation of escape. Furthermore, BAC (bacterial artificial chromosome) transgenes containing *Kdm5c* and its distal (3’ end) boundary inserted at random on the Xi can maintain *Kdm5c* escape status at various insertion sites during mouse embryonic stem cell differentiation [23]. However, in the absence of the distal boundary there is inappropriate spreading of escape from the truncated *Kdm5c* BAC to neighbor genes normally subject to XCI, supporting the idea that insulator elements separate domains of escape from silenced domains. An in vivo transgenic mouse study further shows maintenance of escape after insertion of BACs containing either a mouse (*Kdm5c*) or a human (*RPS4X*) escapee and their flanking neighbor genes into *Hprt*, a gene subject to XCI [24]. More intriguingly, such CTCF-mediated insulation can be achieved by artificial CTCF tethering via CRISPR/dCpf1, which can be exploited to help reactivate *MECP2* on the Xi when combined with CpG demethylation [25]. Consistently, escapees enriched with CTCF binding are often located at the edge of the three-dimensional (3D) model of the Xi but not of the Xa which were built based on allelic chromatin conformation contacts [26]. While these findings strongly suggest that CTCF binding as well as DNA boundaries or other cis elements spatially regulate escape, the molecular mechanisms remain elusive.

In this study we systematically examined allele-specific CTCF binding patterns and epigenetic features at constitutive and facultative escapees in Patski cells and in adult mouse F1 hybrid tissues, using ChIP-seq, CUT&RUN, and ATAC-seq analyses, as well as chromatin conformation analyses. We found that escapees on the Xi are often located inside domains of ~ 30–800 kilobases (kb), marked by convergent arrays of CTCF binding sites. In addition, strong and divergent CTCF binding sites are often located at a boundary between escapees and adjacent genes subject to XCI. To functionally test the role of CTCF binding in escape, we applied an allele-specific CRISPR/Cas9 approach to delete or invert a strong CTCF binding site at the boundary proximal to the promoter of the facultative escapee *Car5b* on the Xi or Xa in Patski cells. We also explored the effects of disrupting the condensation and heterochromatic environment of the entire Xi on escape levels. These studies affirm the contributions of boundary elements such as CTCF binding, and of histone modifications and chromatin condensation in modulation of escape.

## Results

### Distribution of CTCF binding around escape genes

CTCF binding and epigenetic features around escapees were systematically annotated in interspecific mouse systems with completely skewed XCI in which Xi and Xa alleles can be identified based on SNPs (single nucleotide polymorphisms) between C57B/6 J (B6) and *Mus spretus* (*sp*) (see Methods). These systems include the embryonic fibroblast line Patski with the B6-Xi and brain tissue from F1 hybrid female mice with the *sp*-Xi. We previously reported the inactivation status of genes by RNA-seq, together with allelic profiles of RNA polymerase II and CTCF binding obtained by ChIP-seq, and chromatin accessibility determined by ATAC-seq in these two systems [16, 27]. Here, all 876 CTCF peaks retained on the Xi in Patski cells were re-examined for motif score and orientation using CTCFBSDB2.0 [28] (Additional file 2: Table S1). We specifically focused on eight regions containing either constitutive escapees (*Ddx3x*, *Kdm6a*, *Eif2s3x*, *Xist*, *Pbdc1*, and *Kdm5c*) or tissue-specific facultative escapees (*Shroom4*, *Car5b*) that both escape XCI in Patski cells but not in brain (Additional file 1: Fig. S1A, B, Additional file 2: Table S2). These eight escapees were selected because they show a significant level of expression from the Xi in the tissue(s) where they escape XCI based on allelic expression and RNA polymerase II profiles, using a cut-off for the expression ratio of Xi/Xi + Xa ≥ 0.1, with expression levels ≥ 5 TPM/RPKM (transcripts per million/reads per kilobase of transcript per million reads mapped) (Additional file 1: Fig. S1A, B). Note that genes with low escape levels often lack multiple CTCF binding sites flanking them, suggesting that CTCF may not be involved in regulation of low levels of escape, which could represent leaky or insufficient silencing [18, 19]. By the criteria listed above, no genes were classified as facultative escapees in mouse brain, while four genes (*Mid1*, *Asmt*, *Atp6ap1* and *1810030O07Rik*) were classified as facultative escapees in addition to *Car5b* and *Shroom4* in Patski cells. However, these four genes were not included in our analysis due to the following observations. *Mid1* spans the boundary of the pseudoautosomal region (PAR), and *Asmt* is inside the PAR, but the arrangement of these two genes differs between B6 and *Mus spretus*, which complicates survey of CTCF binding patterns on the Xi and Xa [29]. In the case of *Atp6ap1* RNA-seq reads map through the gene body on the Xa allele, but only at the 5’ end of the Xi allele based on a single SNP, which suggests mis-assigned reads due to an incorrect SNP and absence of escape (Additional file 1: Fig. S1C). While *1810030O07Rik* shows evidence of escape [30], we found no evidence of CTCF binding sites in the regions covering the gene and its neighbors subject to XCI on either the Xi or the Xa (Additional file 1: Fig. S1D).

**Fig. 1:**
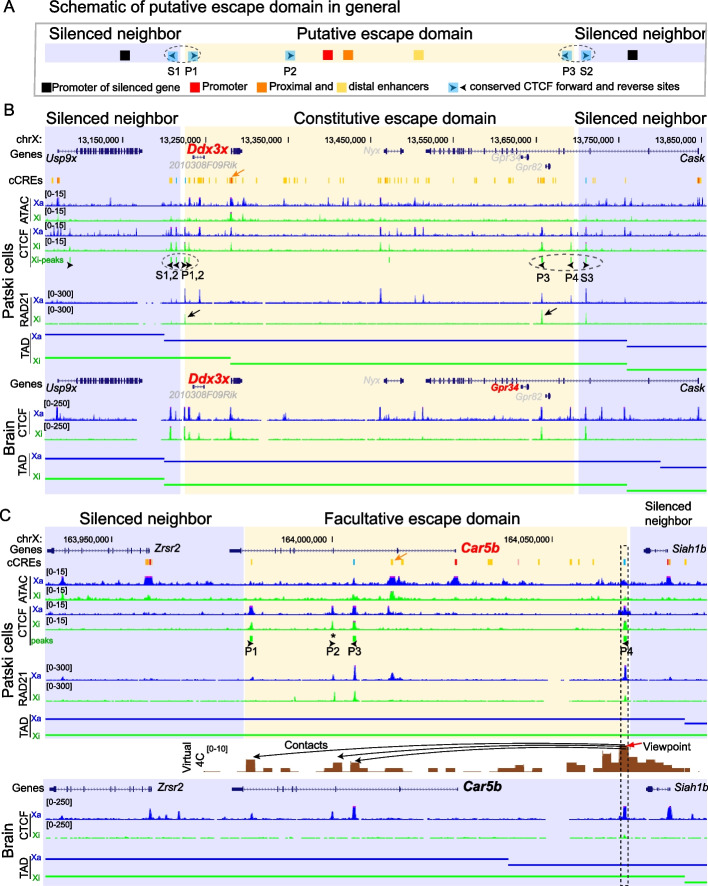
Escape domains at the constitutive escape gene *Ddx3x* and facultative escape gene *Car5b*. **A**. Schematic of a putative escape domain (pale yellow) flanked by domains of silencing (blue). Convergent CTCF sites (forward P1 and P2 and reverse P3) are hypothesized to help form loop(s) that contain the promoter (red square), proximal and distal enhancers (orange and yellow squares) in a putative escapee domain. Conserved CTCF sites are labeled by a cyan square with the motif orientation indicated by arrowhead. Adjacent divergent CTCF sites (S1/P1 and S2/P3) that straddle the boundaries of the escape domain (marked by dotted ovals) would insulate the domain from silenced neighbors which promoter is labeled as a black square. **B**. UCSC browser view of the constitutive *Ddx3x* domain in Patski cells and in mouse brain. Allelic profiles of ATAC-seq reads, CTCF and RAD21 ChIP-seq reads and TADs on the Xa (blue) and Xi (green) are shown. The boundaries between the putative escape domain (yellow) and its flanking silent regions (blue) are marked by several CTCF binding sites with divergent orientation (dotted ovals). Inside of the escape domain convergent arrays of CTCF binding sites and RAD21 peaks suggest potential loop interactions mediated by forward motifs P1, P2, and reverse motifs P3, P4 (black arrowheads). Only CTCF Xi-peaks with a conserved motif score ≥ 10 from CTCFBSDB are marked. The *Ddx3x* escape domain approximately overlaps with an Xi TAD. ENCODE candidate *cis*-regulatory elements (cCREs) combined for all mouse cell types are shown for promoters (red), enhancers (orange), and conserved CTCF sites (cyan). A strong putative proximal enhancer cCRE of *Ddx3x* is marked by an orange arrow based on the highest Xi-H3K27ac enrichment close to this escapee (Additional file1: Fig. S3). Profiles of CTCF ChIP-seq reads and TADs in mouse brain are shown. Note that *Gpr34,* which is not expressed in Patski cells, escapes XCI in brain (Additional file1: Fig. S2G). Genes known to escape XCI are labeled in red, genes subject to XCI, in black, and genes that are not expressed or not assessable due to lack of SNPs, in grey. **C**. Same analysis for the facultative escapee *Car5b* in Patski cells and mouse brain. CTCF binding site P4 is located between *Car5b* and its inactivated neighbor *Siah1b*. Three CTCF binding sites are located within the body of *Car5b* (P1, P2 and P3). While no CTCF peak was called at P2 on the Xi using our cutoff for peak calling on the CTCF profile from ChIP-seq (star), CTCF binding regions P1 and P2 include strong CTCF forward motifs, and P2 also binds RAD21 on the Xi, suggesting that P1, P2 and P3 are important anchor sites. In support of this, the virtual 4C contact plot derived from Hi-C data in Patski cells at 1 kb resolution using a 5 kb window around CTCF peak P4 (marked by red arrow) as a viewpoint reveals contacts that overlap peaks P1, P2 and P3, suggesting looping events involving three regions within the body of *Car5b*. There is no correlation with the TAD structure. Note that the *Car5b* promoter and distal enhancer marked by ATAC-seq peaks and ENCODE cCREs (red and yellow bars) lack strong CTCF peaks on the Xi. A strong putative distal enhancer cCRE of *Car5b* is marked by an orange arrow based on the highest Xi-H3K27ac enrichment close to this escapee (Fig. 4). Importantly, there is no or very little CTCF binding on the Xi in brain where *Car5b* does not escape XCI

**Fig. 2:**
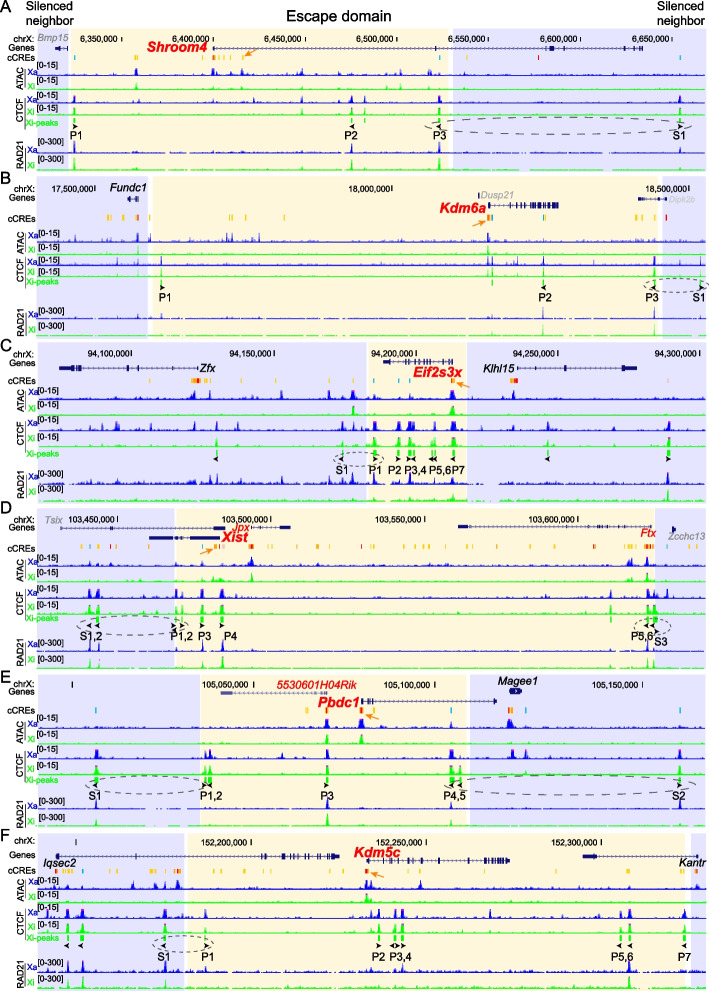
Escape domains at the facultative escape gene *Shroom4* and at the constitutive escape genes *Kdm6a, Eif2s3x, Xist, Pbdc1, and Kdm5c*. UCSC browser views of domains around the facultative escapee *Shroom4* (**A**) and the constitutive escapees *Kdm6a*, *Eif2s3x, Xist, Pbdc1*, and *Kdm5c* (**B-F**). Allelic profiles of ATAC-seq reads and CTCF and RAD21 ChIP-seq reads on the Xa (blue) and Xi (green) are shown. Nine of 16 boundaries between the putative escape domain (yellow) and its flanking silent regions (blue) are marked by CTCF binding sites with divergent orientation (dotted ovals). Inside of the escape domain convergent arrays of CTCF binding sites and RAD21 peaks suggest loop interactions mediated by forward motifs and reverse motifs (black arrowheads). ENCODE cCREs are shown for promoters (red), enhancers (yellow/orange), and conserved CTCF sites (cyan). A strong putative proximal/distal enhancer cCRE of an escapee is marked by an orange arrow based on the highest Xi-H3K27ac enrichment close to this escapee (Additional file1: Fig. S3). Genes known to escape XCI are labeled in red, genes subject to XCI, in black, and genes that are not expressed or not assessable due to lack of SNPs, in grey. See Additional file 1: Fig. S2A-F for profiles in mouse brain, and Fig. 1 for the profiles at the constitutive escapee *Ddx3x* and facultative escapee *Car5b* in Patski cells

CTCF binding site strength is best described by both motif score and significant binding signal (e.g., ChIP-seq peak height) [31]. Thus, we selected CTCF Xi-peaks that have a motif score of at least 10 and marked their forward or reverse orientations around the selected eight escapees (black arrowheads in Fig. 1, Fig. 2). The promoter and enhancer(s) of all eight escapees marked by the location of both known candidate cis-regulatory elements (cCREs) from mouse ENCODE [32] and allele-specific high chromatin accessibility (ATAC-seq peaks), were found to be flanked by convergent arrays of CTCF binding sites (Fig. 1, Fig. 2). It has been shown that pairs of convergent CTCF sites represent the strongest loop anchors, while divergent CTCF sites are much less favored for mediating loops [33, 34]. Furthermore, clustered CTCF binding sites often define and maintain conserved topologically associated domains (TADs) [31, 35]. The convergent CTCF binding sites observed at escapees may serve as strong anchors of chromatin loops mediated by cohesin to establish escape domains. Thus, we defined each putative escape domain as the largest genomic region that encompasses the promoter and predictive enhancer(s) of a given escapee(s) and has its borders marked by a pair of convergent CTCF-binding sites on the Xi (i.e., the forward site as the left border and the reverse site as the right border), which would insulate the escape region from the promoter of neighbor genes subject to XCI (Fig. 1A). Each putative domain at the eight escapees has at least three strong CTCF binding sites, with forward ones at the left section and reverse ones at the right section. In support of this, RAD21, a cohesin core subunit, shows binding peaks that overlap with CTCF binding peaks at or near 12 of the 16 borders of escape domains (Fig. 1, Fig. 2). In addition, strong and adjacent CTCF binding sites arranged in a divergent back-to-back motif configuration (P and S sites) are often located at the boundaries between escapees and neighbor genes subject to XCI, suggesting insulation of the escape domain from the silenced domain (10/16 boundaries; dotted circles in Fig. 1, Fig. 2). Nine of 16 boundaries contain conserved CTCF binding sites overlapping with mouse cCREs (cyan) as classified by ENCODE [32]. The CTCF binding sites at the remaining seven boundaries were only present in Patski cells and/or a few ENCODE mouse cell lines.

**Fig. 3:**
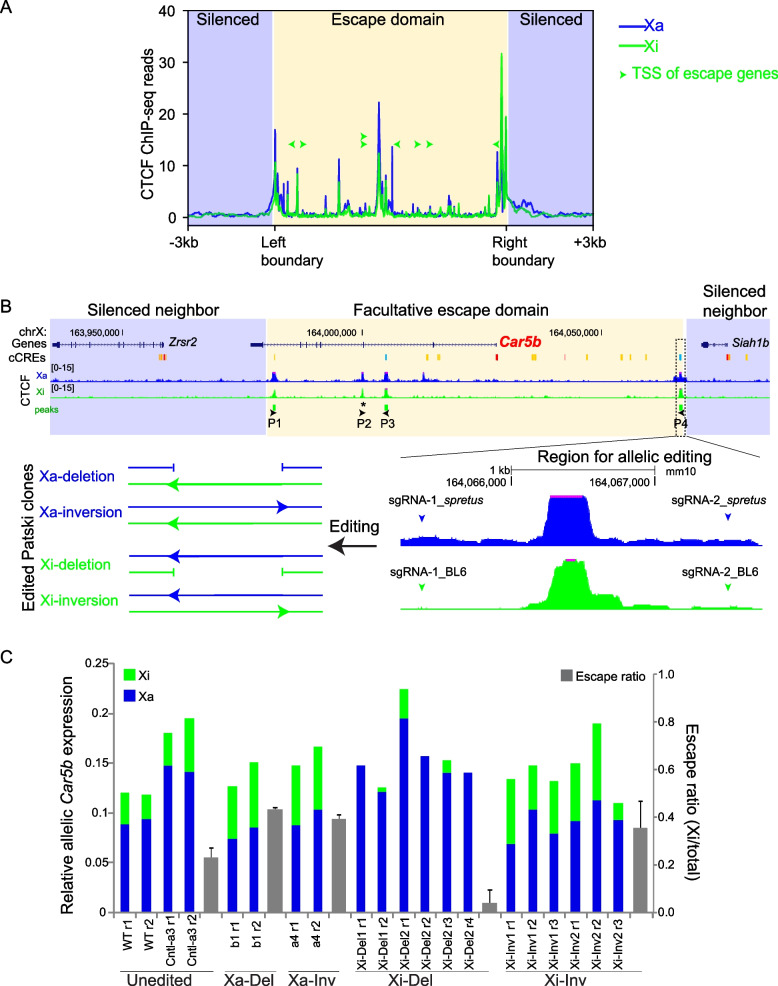
The CTCF boundary region P4 between *Car5b* and *Siah1b* contributes for *Car5b* escape. **A** Metafile analysis of CTCF allelic ChIP-seq reads at eight escape domains in Patski cells shows a similar CTCF-binding pattern between the Xa (blue) and Xi (green) (see also Fig. 1, Fig. 2). The TSS (transcriptional start sites) of escapees, which are located between CTCF peaks are marked by green arrowheads that indicate the direction of transcription. **B** Schematic of allelic CRISPR/cas9 editing of the 2 kb CTCF peak region P4 at *Car5b* to derive deletions and inversions either on the Xi or Xa. As shown on the enlarged region a pair of B6-specific or *spretus*-specific single-guide RNAs (sgRNAs) was used for editing (see Additional file 1: Fig. S5, Additional file 2: Tables S3, S5). Following editing, clones with Xa-deletion, Xa-inversion, Xi-deletion and Xi-inversion with black arrowheads indicating the orientation of CTCF binding sites at P4 were isolated. **C**. Allelic *Car5b* expression from the Xi (green bar) and the Xa (blue bar) measured by quantitative PCR performed on pre-amplified RT-PCR products with or without (mock) ApaI digestion, which specifically cleaves the B6 allele on the Xi. Relative Xi- and Xa-expression levels are shown in unedited controls (WT and cloned Patski cells in which editing failed), one clone with deletion on the Xa (Xa-Del), one clone with inversion on the Xa (Xa-Inv), two independent clones with deletion on the Xi (Xi-Del), and two independent clones with inversion on the Xi (Xi-Inv). At least two biological replicates were tested for WT Patski cells and for each cloned line. Escape levels of *Car5b* (grey bar) calculated from the Xi/total expression ratios are shown as means ± SEM. Only the Del-Xi lines show a significant decrease in escape levels, as compared to any other group (*P* < 0.0005, unpaired two-tail *t*-test). Note that Xi-specific expression ranges between 20–40% of total *Car5b* expression

Interestingly, the CTCF binding sites defining the putative escape domains of the eight escapees are present on both the Xa and Xi (Fig. 1, Fig. 2), while the rest of the Xi shows a chromosome-wide depletion of CTCF binding compared to the Xa [16]. Indeed, metafile analysis of allelic CTCF ChIP-seq signals shows a similar CTCF-binding pattern between the Xa and Xi at escape domains in Patski cells (Fig. 3A). Consistent with previous studies [16, 22], this indicates that CTCF binding is retained on the Xi, which could contribute to escape by both insulating escapees from the heterochromatic environment of the Xi and enhancing gene expression. While the CTCF binding sites at domains containing the constitutive escapees *Ddx3x*, *Kdm6a*, *Eif2s3x*, *Xist*, *Pbdc1*, and *Kdm5c* are maintained on the Xi both in Patski cells and in adult mouse brain, those at domains containing the facultative escapees *Car5b* and *Shroom4* are only present on the Xi in Patski cells where the genes escape XCI (Fig. 1, Fig. 2, Additional file 1: Fig. S2A-F). Five of the eight escape domains contain a single escapee, but large escape domains can contain additional escapees. For example, the *Ddx3x* domain contains *Gpr34* that is macrophage/microglia specific and is not expressed in Patski cells, thus precluding defining its escape status in this cell type. However, allelic RNA-seq analysis in mouse brain shows a low level of *Gpr34* escape (Additional file 1: Fig. S2G), which confirms a previous study demonstrating that *Gpr34* escapes XCI in frontal cortex containing microglia [36]. This illustrates the potential for gene activation within an escape domain insulated by CTCF when tissue-specific transcription factors (TFs) are available. The *Xist* domain (150 kb) contains two other escapees, *Jpx* and *Ftx*, both implicated in the process of XCI, while the *Pbdc1* domain contains a long noncoding RNA gene *5530601H04Rik*, also known to escape XCI (Fig. 2D, E) [37–39].

We conclude that escape domains are marked by a specific spatial pattern of CTCF binding sites present on both the Xa and Xi. For constitutive escapees this configuration remains unchanged in Patski cells and adult mouse brain, but for facultative escapees CTCF binding is only present in cells where the gene escapes XCI.

### Characterization of escape domains

We next examined the relationship between the putative escape domains and TADs. We found that the escape domains are usually smaller than the TADs we previously defined on the Xi using Hi-C allelic contacts at 520 kb windows with 40 kb resolution (Fig. 1, Additional file 2: Table S2) [27]. Thus, escape domains, which size is usually less than 200 kb, are often located inside TADs and may represent allelic sub-TAD structures or loops (Additional file 2: Table S2). Two exceptions are the *Ddx3x* domain that measures ~ 500 kb and overlaps with a known TAD in both Patski cells and brain (Fig. 1) [27], and the *Kdm6a* domain that measures ~ 800 kb and overlaps a very large TAD in Patski cells and a smaller one in brain.

The spatial distribution of CTCF binding sites was then mapped relative to the location of the promoter and enhancer(s) of each of the eight escapees within their putative escape domain. For four of the escapees, *Shroom4*, *Xist*, *Pbdc1*, and *Car5b,* CTCF binding sites with convergent motifs flank the promoter and enhancer(s) but not the 3’ end of the gene (Fig. 1, Fig. 2). Rather, the distal border of the escape domain marked by one of the convergent CTCF sites is located within an intron of each of these genes, suggesting the formation of loops that mainly insulate the promoter and enhancer(s). Accordingly, the TSS (transcriptional start site, i.e., the center of the promoter marked by a green arrowhead in Fig. 3A) of each escapee is often located between CTCF peaks, which would help insulate this critical region for access to TFs that mediate promoter-enhancer interactions and activate genes. Notably, it has been suggested that the transcriptional machinery can travel through chromatin boundaries for transcription elongation, consistent with the idea that a loop that engages a region within a gene would not impede transcription [31].

Allelic chromatin accessibility and histone modifications including H3K27ac and H3K27me3 were mapped at each escape domain. Except for *Xist*, which is only expressed from the Xi, we found that chromatin accessibility is maintained but at a reduced level at all eight escape domains on the Xi compared to the Xa (Fig. 1, Fig. 2). Similarly, enrichment in the active enhancer mark H3K27ac is lower on the Xi (Additional file 1: Fig. S3). This is consistent with reduced levels of RNA polymerase II and lower expression of escapees from the Xi versus the Xa [16, 18, 30, 40]. In contrast, there is no or minimal chromatin accessibility and H3K27ac enrichment at silenced neighbors on the Xi. The repressive histone mark H3K27me3 is depleted throughout all eight escapees on the Xi but retained at regions close to the boundaries of three escape domains (*Ddx3x*, *Kdm6a*, and *Kdm5c*) (Additional file 1: Fig. S3). Thus CTCF-mediated insulation may prevent long-range spreading of H3K27me3 into escape domains but may not prevent partial spreading across the boundary (Additional file 1: Fig. S3B, C, G). The strong CTCF binding peaks at the boundaries of escape domains often lack chromatin accessibility as shown by the absence or low level of ATAC-seq peaks on both the Xi and the Xa (Fig. 1, Fig. 2), suggesting a tight chromatin structure protected by strong CTCF binding. Conversely, the ATAC-seq peaks at the promoter and enhancer(s) of escapees (e.g., *Car5b*, *Pbdc1*, *Kdm5c*) often correlate with the absence or low level of CTCF binding. This is consistent with the TSS of escapees being usually located between CTCF peaks (Fig. 3A), and is in line with previous findings that YY1, not CTCF, is a major regulator of promoter-enhancer interactions [41].

In summary, CTCF binding sites are often located proximal to the promoter and enhancer(s) of escapees, suggesting that insulation of the promoter and enhancer regions allows escape. Retention of chromatin accessibility and of the active histone mark H3K27ac as well as depletion of the repressive histone mark H3K27me3 are associated with expression of escapees on the Xi, albeit at a reduced level compared to the Xa.

### Contribution of CTCF insulation to Car5b escape from XCI

To test the role of CTCF in regulation of escape from XCI, we focused on the tissue-specific facultative escapee *Car5b* (Fig. 1B). *Car5b/CA5B* encodes a highly conserved mitochondrial carbonic anhydrase expressed in multiple human and mouse tissues with increased expression during development and high expression in kidney (Additional file 1: Fig. S4A-B). In human *CA5B* escapes from XCI in most tissues [14]. In contrast, *Car5b* is subject to XCI in mouse adult tissues including brain, spleen, heart, kidney, liver and ovary as well as in MEFs (mouse embryonic fibroblasts) (Additional file 1: Fig. S4C, D). However, *Car5b* escapes XCI in Patski cells originally derived from E18.5 embryonic kidney, which provides a cell line system for editing the escape domain. We identified four CTCF binding sites, P1-4 in the *Car5b* domain, which are present on the Xi in Patski cells but not in brain, suggesting that CTCF-mediated insulation may protect *Car5b* escape in these cells (Fig. 1C) [16].

The CTCF binding site P4 is located at the boundary between *Car5b* and its neighbor *Siah1b* that is subject to XCI. This is a conserved CTCF binding site as predicted by cCRE, which includes a high reverse motif score of 26 (chrX:164,066,195–164066766; Additional file 2: Table S1). The P4 region centered around the CTCF motif with 25nt up- and downstream has an identical sequence in B6 and *sp*, ruling out any genetic effect that could explain differential binding on the BL6 Xi in Patski cells versus the *sp* Xi in brain. The three other CTCF binding sites (P1-3) are located within *Car5b* introns. Virtual 4C (circular chromosome conformation capture) analyses based on Hi-C (high-throughput chromosome conformation capture) contacts show interactions between P4 and the three CTCF sites (P1-3) located within *Car5b* (Fig. 1C) [27]. Note that limited contacts in this region impede allele-specific virtual 4C analysis. We hypothesized that formation of an insulated loop domain via CTCF-mediated interactions may contribute to *Car5b* escape, which is supported by our findings of RAD21 binding at P2-4. To directly test the role of P4 we deleted or inverted it in wild-type (WT) Patski cells using allele-specific sgRNAs for CRISPR/Cas9 editing (Fig. 3B, Additional file 1: Fig. S5A, Additional File 2: Table S3). Two independent clones were derived for each editing type on the Xi (Xi-Del1, Xi-Del2, Xi-Inv1, Xi-Inv2), and one clone for each editing type on the Xa (Xa-Del1, Xa-Inv1) (Additional File 2: Table S3). Correct editing of the targeted alleles and retention of the intact CTCF site on the unedited alleles were confirmed by PCR and Sanger sequencing (Additional file 1: Fig. S5B, C, Additional file 2: Table S3). The intact alleles in each clone served as internal controls for functional analyses.

**Fig. 4:**
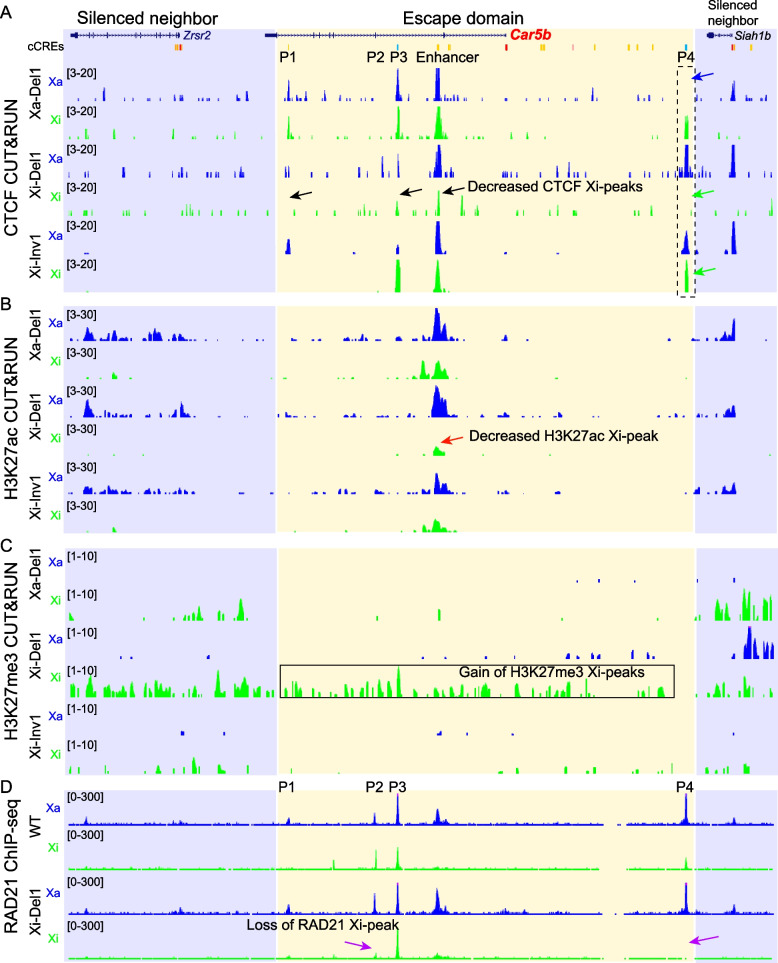
CTCF binding protects *Car5b* from heterochromatic marks on the Xi. **A** UCSC browser view of allelic profiles of CUT&RUN reads for CTCF at the *Car5b* escape domain (yellow) and surrounding silent regions (blue) in Xa-Del1, Xi-Del1 and Xi-Inv1. The expected loss of CTCF binding due to editing is seen at the P4 region (blue and green arrows). In Xi-Del1 the CTCF binding peaks at P1 and P3 (black arrows) are reduced consistent with interactions between P4/P1 and P4/P3. No apparent changes were observed in Xa-Del1 or Xi-Inv1 cells. **B** Allelic profiles obtained by CUT&RUN for H3K27ac show a decrease at the enhancer on the Xi (red arrow) in Xi-Del1. No changes were observed in the other lines. **C** Allelic profiles for H3K27me3 show a strong enrichment of the repressive mark (box) throughout the *Car5b* domain in Xi-Del1 compared to other lines, suggesting that CTCF functions as an insulator to protect *Car5b* escape. **D** Allelic RAD21 profiles obtained by ChIP-seq in WT and Xi-Del1 cells show a loss of RAD21 binding at the edited P4 region and also a decrease at the P2 region (purple arrows) on the Xi in Xi-Del1. No changes were observed on the Xa

Allelic expression analyses of exon-specific transcripts based on B6-specific ApaI digestion [30] show that escape levels of matured *Car5b* transcripts from the Xi are abolished or strongly reduced following deletion of P4 in Xi-Del1 and Xi-Del2, compared to WT cells or to a clone subjected to the editing process, but with no editing of P4. In contrast, inversion of this same region in Xi-Inv clones has no effects on *Car5b* escape (Fig. 3C, Additional file 1: Fig. S6A, B). To test effects of P4 editing on *Car5b* nascent transcripts from the Xi, we designed allelic pairs of primers targeting the intron regions that overlap with P1, P2 and an enhancer region identified by ATAC-seq peaks, H3K27ac peaks, and a cCRE (Fig. 1C, Fig. 4A, Additional file 1: Fig. S7A)*.* Following validation of these primers using PCR on species-specific genomic DNA, we determined that escape levels of nascent *Car5b* transcripts are significantly reduced in Xi-Del clones, with the strongest effect in Xi-Del1, but are unchanged in Xi-Inv clones compared to WT (Additional file 1: Fig. S7, S8). No reactivation of the neighbor gene *Siah1b* was observed in any condition, and no change in *Car5b* expression was observed when P4 was deleted or inverted on the Xa, suggesting that this CTCF binding site is not essential for *Car5b* expression (Fig. 3C, Additional file 1: Fig. S6B-D). Thus, CTCF binding at the boundary region P4 may specifically contribute to enhancing *Car5b* escape from XCI.

Next, we generated allelic profiles for CTCF, H3K27ac, and H3K27me3 by CUT&RUN in Xi-Del, Xi-Inv, and Xa-Del lines, and for RAD21 by ChIP-seq in WT and Xi-Del lines. Deletion of P4 on the Xi results in reduced levels of CTCF and H3K27ac and in increased levels of H3K27me3 over the *Car5b* escape domain on the Xi, consistent with loss of looping and escape (Fig. 4, Additional file 1: Fig. S9). Histone modification changes on the Xi are more pronounced in Xi-Del1, consistent with a greater loss of escape, compared to Xi-Del2 (Fig. 4, Additional file 1: Fig. S9B, C). Indeed, spreading of the silencing mark H3K27me3 is prominent throughout the whole escape domain (~ 85 kb) in Xi-Del1 (Fig. 4C, Additional file 1: Fig. S9C). No apparent changes were found when P4 is inverted on the Xi or deleted on the Xa (Fig. 4).

Two of the three sites (P1, P3) with reduced CTCF binding within the *Car5b* locus in Xi-Del1 overlap with chromatin contact regions identified by virtual 4C based on Hi-C (Fig. 1C, Fig. 4A) [27]. The third site with reduced CTCF binding overlaps with an enhancer (Fig. 1B, Fig. 4A). In Xi-Del2 only P1 and the enhancer showed reduced CTCF binding, suggesting a different chromatin configuration in that clone, which may explain differences in *Car5b* expression between clones (Additional File 1: Fig. S9A). Note that CTCF binding overlapping with the enhancer is only captured by CUT&RUN that can detect both bound regions and regions located in proximity of a binding site [42]. However, ChIP-seq, a method that can only detect bound regions, did not reveal any CTCF signals around the enhancer on the Xi, but there were some weak signals adjacent to the enhancer on the Xa, suggesting that the enhancer is not directly bound by CTCF but is located near a CTCF binding region (Fig. 1C, Fig. 4A, Additional File 1: Fig. S9A). Due to limited SNP reads from CUT&RUN, P2 could not be evaluated for allelic changes. However, by ChIP-seq in Xi-Del1 we observed loss of RAD21 at site P2 that contains a forward CTCF motif and thus could mediate a smaller loop (Fig. 1C, Fig. 4D).

To complement genome-wide ChIP-seq and CUT&RUN analyses, we performed ChIP for CTCF, RAD21 and H3K27ac in WT, Xi-Del1, Xi-Del2, and Xi-Inv1 followed by allelic PCR using B6-specific primer pairs targeting P1, P2, the enhancer, and P4 (see Additional file 1: Fig. S7). In both WT and Xi-Inv1 the strongest CTCF and RAD21 enrichment was detected at P4, as compared to P1, P2 and the enhancer, consistent with ChIP-seq and CUT&RUN results (Additional file 1: Fig. S10). As expected, no Xi enrichment of any marks was detected at P4 in the Xi-Del clones. The second strongest CTCF enrichment was detected at P2 where a 10–20% decrease in CTCF binding was confirmed in Xi-Del1&2, but not in Xi-Inv1 compared to WT cells (Additional file 1: Fig. S10). CTCF enrichment at P1 and the enhancer and RAD21 enrichment at P1, P2 and the enhancer were too weak for evaluation of meaningful changes among clones. However, strong H3K27ac enrichment was observed at the enhancer, with a clear decrease in Xi-Del1 compared to WT, consistent with loss of *Car5b* escape. Note that some changes observed by ChIP-seq and CUT&RUN (e.g., effects at P1) were not detected by ChIP-PCR, which could be due to different sensitivity between methods.

**Fig. 5:**
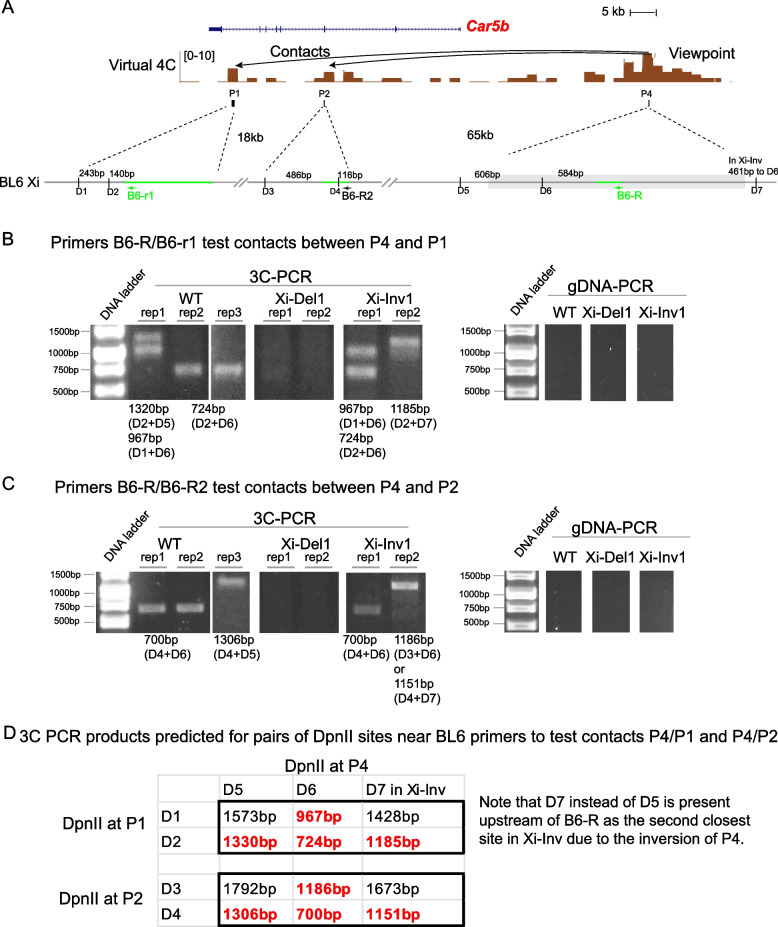
3C-PCR detects Xi-specific chromatin contacts within the *Car5b* escape domain in WT and Xi-Inv. **A**. Schematic of the *Car5b* region indicating the location of B6-Xi primer pairs. B6-R for P4 and B6-R2 for P2 are the same primers shown in Additional file 1: Fig. S7A. Additional B6-Xi primer (B6-r1 for P1) were designed using validated SNPs. For each region, the two closest DpnII sites (D) upstream of each primer are marked together with the distance between the primer and the closest DpnII site and between two DpnII sites. DpnII sites are numbered D1-D7. Note that due to the inversion of P4, D7 rather than D5 is the second closest site upstream of B6-R in Xi-Inv. The deleted or inverted P4 region (1864 bp) is indicated as a gray box. **B**, **C**. 3C-PCR was performed using the selected Xi primer pairs shown in (**A**) to detect potential chromatin contacts between P4 and P1 (**B**) and between P4 and P2 (**C**). PCR products show the presence of chromatin contacts in WT and Xi-Inv, but not in Xi-Del1, which was used to exclude the possibility of any PCR products from contacts with P4 on the *sp*-Xa. Two biological replicates of 3C per genotype were performed except for WT with one additional replicate. Note that PCR products from chromatin contacts can vary in size, due to ligation of different DpnII sites from incomplete digestion, as predicted below the gels and in (**D**). PCR using genomic DNA (gDNA) from WT, Xi-Del1 and Xi-Inv1 shows no products from these B6-specific primer pairs, indicating the specificity of 3C-PCR. **D**. Summary of 3C products predicted for pairs of DpnII sites upstream of the primers within the loci of interaction (P4/P1, P4/P2). The two closest DpnII sites upstream of each primer are shown. Incomplete digestion results in different sizes of ligation products. Those detected here by 3C-PCR are highlighted in red

To confirm chromatin contacts within the *Car5b* escape domain on the B6 Xi inferred by virtual 4C in WT cells (Fig. 1C) and to test their presence or absence in Xi-Inv cells, we performed in situ chromosome conformation capture (3C) with DpnII digestion and proximity ligation (see Methods). PCR using B6-specific primer pairs, which were verified to not amplify unligated genomic DNA, was done on ligated products to detect any interactions between P4 and P1 and between P4 and P2 on the Xi in WT and Xi-Inv1 (Fig. 5). We clearly detected PCR products consistent with Xi chromatin contacts between P4 and P1 and between P4 and P2 in WT and Xi-Inv1 but not in Xi-Del1 (Fig. 5B-D). This is consistent with virtual 4C. As an additional control we excluded the presence of PCR products from any contacts with P4 on the *sp*-Xa in Xi-Del1. Note that P3 with a reverse CTCF motif was not tested here since this site does not form a convergent pair with P4 in WT cells and is not or only slightly changed in terms of CTCF and RAD21 enrichment upon Xi-deletion of P4 (Fig. 4A, D, Additional file 1: Fig. S9A).

Our allelic analysis of gene expression and epigenetic features in cell lines with allele-specific deletions or inversions of a CTCF boundary strongly supports the role of CTCF in insulating the *Car5b* escape domain on the Xi via formation of contacts or loops that protect *Car5b* escape.

### Escape genes are sensitive to disruptions of the Xi-specific heterochromatic environment

Our findings of reduced chromatin accessibility, H3K27ac enrichment, and expression levels of escapees on the Xi compared to the Xa suggest an interplay between heterochromatin environment and chromatin insulation to modulate escape levels due to the activity of additional chromatin factors. To identify such factors, we took advantage of two mutant cell lines we previously derived in which the overall structure of the Xi and its enrichment in the repressive histone mark H3K27me3 are altered. Indeed, disruption of the Xi compact heterochromatic structure can be induced by deletion of the *Dxz4* locus (Del-Hinge) [27], and depletion of *Firre* RNA leads to loss of H3K27me3 on the Xi [43]. We also examined the role of DNA methylation, an important epigenetic regulator of XCI [44].

**Fig. 6:**
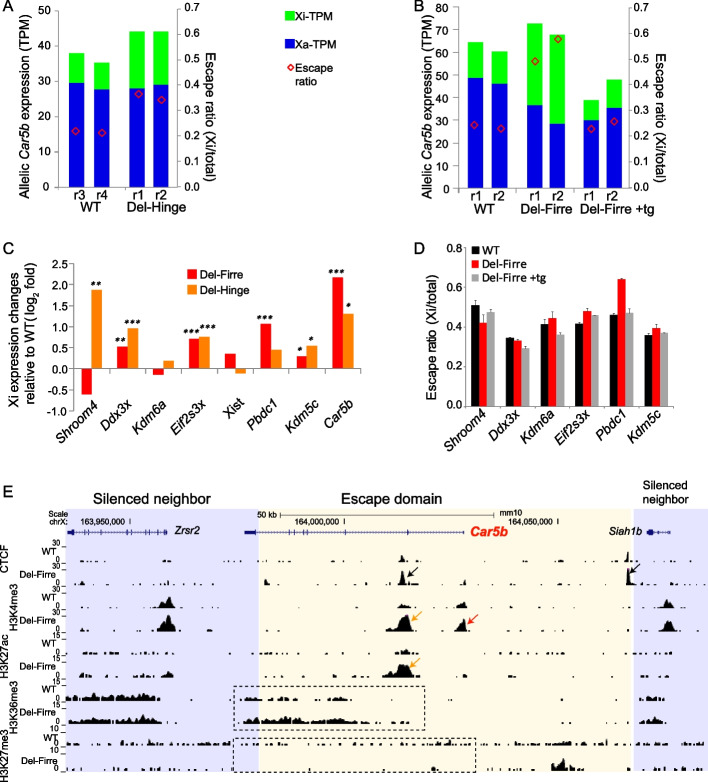
The chromatin environment constrains expression levels of escape genes on the Xi. **A** Disruption of the Xi condensed structure by deletion of the *Dxz4* locus on the Xi (Del-hinge) increases *Car5b* expression on the Xi. Allelic expression levels (TPM, transcripts per million) for the Xa (blue) and Xi (green) are shown together with escape ratios (red diamond). RNA-seq data from [27]. **B** Depletion of *Firre* RNA in Patski cells by deletion of the lncRNA on the Xa (Del-Firre), which is known to cause a loss of H3K27me3 on the Xi, increases escape levels of *Car5b.* This effect can be reversed using a *Firre* cDNA transgene (Del-Firre + tg). Same analysis as in A. RNA-seq data from [43]. Also see Additional file 2: Table S4. **C** Deletion of the *Dxz4* locus and depletion of *Firre* RNA result in an increase of Xi-expression levels 7/8 escapees and 6/8 escapees, respectively, as shown by Xi-expression fold changes compared to WT. Fold changes and adjusted *p*-values (* < 0.1, ** < 0.05, *** < 0.01) were derived from allelic DESeq2 analysis of RNA-seq data [27, 43]. **D** Increased escape levels of five escapees, except *Shroom4*, upon *Firre* RNA depletion (Del-Firre) are restored by ectopic expression of a *Firre* cDNA transgene (Del-Firre + tg). Error bar: s.e.m. Also see Additional file 2: Table S4. Note that increased escape levels are more apparent in Del-Firre versus Del-Firre + tg, probably because the transgenic line, unlike WT, is derived from Del-Firre. An unpaired two-tail *t*-test was performed between WT and Del-Firre, between WT and Del-Firre + tg, and between Del-Firre and Del-Firre + tg. Only the escape ratio of *Pbdc1* in Del-Firre is significantly different from that in WT (*p* = 0.002) or Del-Firre + tg (*p* = 0.038). **E.** UCSC browser view of total reads from CTCF, H3K4me3, H3K27ac, H3K36me3, and H3K27me3 CUT&RUN in WT and Del-Firre Patski cells. Note that allelic analysis was not possible due to limited read coverage and short reads (36nt). Considering that expression of *Car5b* on the Xi but not on the Xa is significantly increased in Del-Firre cells as shown by allelic RNA-seq in **B**, we infer that increases in CTCF (black arrows) and in active histone marks at the promoter (H3K4me3; red arrow), distal enhancer (H3K4me3 and H3K27ac; orange arrow), and 3’ region (H3K36me3 for elongation; dotted box) of *Car5b* are probably from the Xi allele. The decrease in H3K27me3 enrichment (dotted box) is also probably from the Xi allele, as suggested by loss of Xi-specific H3K27me3 upon *Firre* depletion [43]

We found that Xi-expression of escapees increases in the two mutant cell lines. In particular, *Car5b* Xi-expression increases 1.6-fold and 2.3-fold following *Dxz4* deletion and *Firre* RNA depletion, respectively (Fig. 6A, B). Interestingly, ectopic expression of a *Firre* cDNA transgene completely restores normal *Car5b* escape levels, consistent with the reversible trans-acting effect of *Firre* RNA on H3K27me3 enrichment on the Xi (Fig. 6B) [43]. For most of the other seven escapees we also found increases in Xi-expression levels (Fig. 6C). Again, escape levels of these genes in Del-Firre can be restored by ectopic expression of a *Firre* cDNA transgene (Fig. 6D, Additional file 2: Table S4), although changes in escape levels are not significant except for *Pbdc1*. Note that *Shroom4* is an exception, with both Xa and Xi levels largely decreased upon *Firre* deletion and restored by the transgene, which impedes the evaluation of the effect of *Firre* depletion on *Shroom4* escape (Fig. 6D, Additional file 2: Table S4). Taken together, our results indicate that the heterochromatic environment of the Xi, including chromatin condensation and H3K27me3 enrichment, normally constrains the levels of expression from escapees on the Xi. Since the escapees are distributed along the whole Xi, these effects appear to be chromosome-wide.

Next, we looked for changes in chromatin accessibility in cells with deletion of *Dxz4* (Del-Hinge), which reveal a moderate increase in accessibility at *Car5b* on the Xi as measured by ATAC-seq (Additional file 1: Fig. S11A), consistent with a modest expression increase (Fig. 6A, C). Since chromatin organization of the Xi becomes more like that of the Xa in Del-Hinge cells [27], we also edited the same CTCF boundary region P4 in Del-Hinge cells to test whether the boundary still helps *Car5b* escape. Indeed, deletion of P4 on the Xi in Del-hinge cells (Xi-Del3) strongly reduces *Car5b* escape, similar to what is observed in Xi-Del1&2 cells derived from WT (Additional file 1: Fig. S11B). Thus, the CTCF boundary helps *Car5b* escape whether the hinge is intact or disrupted, consistent with the notion that *Xist* coating and the silencing mark H3K27me3 are not affected by Del-hinge [27]. The increase in *Car5b* expression from the Xi in Del-Hinge would then be due to lower chromatin compaction of the Xi.

Increased *Car5b* Xi-expression due to loss of *Firre* RNA was further investigated by generating Xi-specific profiles of epigenetic marks in Del-Firre (Fig. 6E). We found an increase in CTCF and in the active marks H3K4me3, H3K27ac, and H3K36me3, and a decrease in the repressive mark H3K27me3, consistent with increased *Car5b* expression. Note that due to limited SNP read coverage allelic analyses were not feasible for CTCF, H3K27ac, H3K36me3 or H3K27me3 (see Methods). However, despite the sparsity of SNP reads, an Xi-specific increase in H3K4me3 enrichment was observed in Del-Firre compared to Del-Firre + tg or WT (Additional file 1: Fig. S12). In addition, since *Car5b* expression from the Xi but not Xa is upregulated after loss of *Firre* RNA (Fig. 6B, C, Additional file 2: Table S4), we speculate that the observed increase in other active marks and decrease in H3K27me3 also occur on the Xi allele.

Escapees often have hypo-methylation of the CpG island at their promoters on the Xi, while genes subject to XCI often have hyper-methylation [14]. However, no CpG island is annotated at the *Car5b* promoter region (UCSC genome browser, [45]), which could explain the absence of DNA methylation at the *Car5b* promoter in frontal cortex where *Car5b* is subject to XCI [36]. However, disruption of DNA methylation could interfere with the heterochromatin environment of the Xi and thus indirectly affect escape. To test whether inhibition of DNA methylation had any effect on *Car5b* escape levels we re-analyzed our previously published RNA-seq data on cells treated by 5-aza-deoxycytidine to inhibit DNA methylation [27]. No effect was found on *Car5b* escape levels with only minor reactivation of neighbor genes *Zrsr2* and *Siah1b* in both WT and Del-hinge cells (Additional file 1: Fig. S13A-C). Interestingly, reactivation levels of these neighbor genes appear to be positively related to their proximity to the boundaries of the escape domain (closer for *Siah1b* than *Zrsr2)*, which might suggest different reactivation potentials due to the influence of chromatin environment from the active *Car5b* escape domain. In addition, escape levels of the neighbor genes were much lower compared to that of *Car5b*, consistent with limited reactivation, which was also shown in a previous report of only ~ 2% of cells with reactivation of a reporter gene after inhibition of DNA methylation [46].

These findings indicate that the 3D structure and the repressive heterochromatic mark H3K27me3 of the Xi, but not DNA methylation, play a role in modulating expression levels of escapees on the Xi.

## Discussion

CTCF is a critical factor for the structural organization of chromatin in the nucleus. In this study we have focused on its role as an insulator of specific regions of the mammalian genome, i.e., regions that contain genes that escape XCI. By mapping CTCF binding sites around genes known to escape XCI we found that a majority of these genes are flanked by CTCF binding sites with convergent motifs. Furthermore, at boundaries between escapees and those subject to XCI we often found strong CTCF binding sites arranged in a back-to-back orientation. These CTCF binding sites may insulate escapees into loops mediated by cohesin to protect them from silencing by allowing access to TFs for gene activation. This is supported by the absence of CTCF binding around two facultative escapees in the tissue in which they are subject to XCI. While these findings are supported by a few transgenic studies [23–25], our study is the first to report the loss of escape following deletion of CTCF binding sites at the *Car5b* gene in a fully differentiated mouse cell line. Our findings of increased expression of escapees in mutant cell lines with disruption of the compact chromatin of the Xi or of its enrichment in H3K27me3 further demonstrate the importance of the overall heterochromatic structure of the Xi in constraining expression of escapees.

Our results show a dramatic loss of *Car5b* escape when the CTCF boundary region P4 is deleted on the Xi in Patski cells, while in brain where the gene does not escape XCI there is no evidence of CTCF binding around *Car5b* on the Xi. In contrast, *Car5b* expression from the Xa where CTCF also binds to region P4 does not depend on this binding, as shown by the absence of gene expression changes after deletion of P4 on the Xa. Previous studies have reported no or limited (often locus-specific) gene expression effects by deletion of a CTCF binding site that disrupts a chromatin loop or by acute depletion of CTCF protein [47, 48]. Thus, CTCF binding may be mainly involved in insulation of chromatin structures and may play a limited role in direct regulation of gene expression [47, 48]. In contrast, YY1 does play a direct role in regulation of promoter-enhancer interactions [41] and is enriched at the TSS of escapees [49]. Our findings of no or weak CTCF binding at promoters and enhancers of escapees confirm these reports. This is also consistent with the recently reported absence of significant difference in predicted probability of CTCF binding at the TSS of escapees versus those subject to XCI in human and mouse [15]. The CTCF binding region P4 located proximal to the *Car5b* contacts CTCF binding regions P1 and P2 located within *Car5b* gene body, as shown by virtual 4C from Hi-C, and by 3C. Thus, the putative insulated domain includes the promoter and enhancer of *Car5b* but lacks its 3’ end, which is sufficient to enhance escape. This configuration, also observed at *Shroom4*, *Xist*, and *Pbdc1*, implies that CTCF-mediated chromatin boundaries apparently do not stall the machinery for transcription elongation, as previously shown [31].

Deletion of the CTCF boundary region P4 causes significant changes in histone modifications associated with changes in gene expression, but there is no spreading of escape into the neighbor gene *Siah1b*. This differs from results of a transgenic study showing that insertion of the escapee *Kdm5c* without its right boundary located inside its neighbor gene *Kantr* (see Additional file: Fig. S2F) causes ectopic escape of genes downstream of the insertion [23]. This may be due to different mouse systems studied (somatic cells in our study and differentiating embryonic stem cells in [23]) or to different mechanisms of regulation among escapees. In particular, regulatory elements associated with constitutive escapees (*Kdm5c*) may differ from those at facultative escapees (*Car5b*). We observe reduced chromatin accessibility, reduced active marks, and persistence of a repressive mark at some regions near the edges of some of the escape domains. This could be related to the highly dynamic nature and short lifespan of loops mediated by CTCF and cohesin [50]. In addition, removal of repressive marks at regions other than the promoter and enhancer(s) of escapees may not be critical for their escape, as the major role of CTCF-mediated insulation could be to provide spatial access to TFs and chromatin modifiers at the promoters and enhancers of escapees within a domain.

We previously reported the position of escapees at the periphery of the 3D structure of the Xi enriched in CTCF binding [26], which is consistent with our current findings of putative insulated domains of escapees on the Xi. Indeed, escapees show increased inter-chromosomal contacts compared to genes subject to XCI [51]. The strength and frequency of loop anchoring via a pair of tandem CTCF binding sites are weaker than via a pair of convergent CTCF binding sites [33, 34]. However, inversion of region P4 on the Xi had no effect on *Car5b* escape, suggesting that chromatin looping, which we confirmed using 3C, is mediated via tandem CTCF binding sites and function as insulation at this site. This is supported by a recent study demonstrating that a pair of dCpf1-fused CTCF molecules can induce insulation and enhance escape of *MECP2*, without any consideration of the orientation of the CTCF binding motifs [25]. However, looping via tandem CTCF motifs could be less maintained/inherited during development and evolution since they appear weaker and less stable than that via convergent CTCF motifs [33, 34].

Our findings of reduced levels of chromatin accessibility and active marks at escape domains on the Xi versus the Xa in WT cells and of an increase in escape levels in cells with disrupted H3K27me3 or de-condensation of the Xi suggest that escape domains are under spatial constraints that limit access to TFs, resulting in lower expression levels from the Xi versus the Xa. Other factors could play a role, for example, escape levels in somatic cells are known to be sensitive to *Xist* RNA levels [52, 53]. Again, different escapees may be differentially regulated. Indeed, loss of H3K27me3 in a *Firre* mutant line resulted in full escape of *Car5b* and *Pbdc1,* but not of other escapees whose expression from the Xi remained lower than on the Xa. We also note that loss of H3K27me3 affect escape levels to a greater extent than Xi de-condensation. The modulation of escape levels could also depend on whether alterations are made before or after the onset of XCI. Indeed, deletion of *Dxz4* in embryonic stem cells prior to the onset of XCI causes loss of escape of the facultative escapee *Mecp2* once the cells are differentiated [54], whereas our findings in Patski cells in which XCI is established levels of escape increase. While CTCF clearly plays an important role in establishing chromatin structure during embryogenesis [55], future studies are needed to track allelic CTCF binding profiles during development, especially at facultative escapees to understand how CTCF binding is retained or lost during the establishment of XCI.

## Conclusions

Our findings support the role of insulation and looping via CTCF binding sites in enhancement of escape from XCI in somatic cells. However, escape domains other than *Car5b* need to be evaluated to generalize this finding. Our study does show that escape from XCI is modulated by the 3D structure of the Xi and its H3K27me3 enrichment but not by DNA methylation at multiple escapees. These findings provide insights for future studies that aim to perturb gene silencing/escape on the Xi to help understand their roles in human health and diseases.

## Methods

### Mouse tissues and cell lines

Female F1 hybrid progeny was obtained by mating C57B/6 J females that carry a deletion of the *Xist* proximal A-repeat (*Xist*^*ΔA*^) (B6.Cg-Xist < tm5Sado > , RIKEN) with *Mus spretus* males (Jackson Labs) as described [16]. Mouse adult tissues and MEFs were collected from female F1 hybrid mice or embryos that inherited a maternal X chromosome with an *Xist*^*ΔA*^. These *Xist*^*ΔA*^/ + tissues and MEFs fail to silence the B6 X chromosome and thus have complete skewing of XCI of the paternal *spretus* X chromosome. Patski cells are fibroblasts previously derived from the 18dpc (days post coitum) embryonic kidney of a F1 hybrid female embryo inheriting an *Hprt*^*BM3*^ mutation on the B6 X chromosome, which was obtained from a cross between a B6 female with an *Hprt*^*BM3*^ mutation and a *spretus* male [56]. Patski cells were selected in Hypoxanthine-Aminopterin-Thymidine (HAT) medium such that the B6 X chromosome with *Hprt*^*BM3*^ needed to be always inactive as verified in previous studies [16, 30]. After selection, Patski cells cultured in regular media were shown to maintain the B6 Xi [30].

### Allele-specific CRISPR/Cas9 editing of Patski cells

Allele-specific CRISPR/Cas9 editing of the CTCF binding region upstream of *Car5b* (P4) was performed in Patski cells as described previously [27]. Allele-specific sgRNAs designed using CHOPCHOP were selected to include B6 or *spretus* SNPs at multiple sites or at the PAM site depending on availability (see Additional file 1: Fig. S5A, Additional file 2: Table S3). DNA oligos to produce sgRNAs were cloned into the CRISPR/Cas9 plasmid pX330 (Addgene). Patski cells (WT and Del-hinge) were transfected with a pair of CRISPR/Cas9 plasmids targeting cut1 and cut2 sites respectively (see Additional file 1: Fig. S5A) using Ultracruz transfection reagents (Santa Cruz), followed by single-cell cloning. PCR together with Sanger sequencing was used to verify specific P4 deletion or inversion of the B6 or *spretus* allele in each clone and to map and verify junction sequences containing B6 SNPs (Additional file 1: Fig S5B, Additional file 2: Table S3). WT Patski cells and clones that had been subjected to transfection but did not carry any deletions or inversions of P4 were used as unedited controls (Ctrl-a3, Dh-a1 and Dh-a7). The sequence of the primers used is in Additional file 2: Table S5.

### Allelic expression analysis using B6-specific restriction enzyme digestion

Allelic expression analysis of *Car5b* was done by B6-specific ApaI digestion as described [30]. In brief, cDNA, obtained by reverse transcription (RT) of RNA prepared from a Qiagen RNAeasy kit, and genomic DNA were amplified by PCR using primers flanking a B6-specific ApaI recognition site in exon 4 of *Car5b*. PCR products were then purified, followed by mock (-ApaI) or ApaI digestion and gel electrophoresis (see Additional file 1: Fig. S6A, B). In Patski cells the appearance of a digested band indicates expression from the B6 allele on the Xi and thus escape from XCI. In contrast, in mouse tissues and MEFs the presence of an undigested band would indicate expression from the *spretus* allele on the Xi and thus escape from XCI (see Additional file 1: Fig. S4C). Allelic expression analysis of *Siah1b* was done similarly using B6-specific BsrI digestion (see Additional file 1: Fig. S6C-D). The primer sequences are listed in Additional file 2: Table S5. To quantify escape levels, quantitative PCR was performed using a SYBR green system for pre-amplified RT-PCR products with mock or B6-specifc ApaI digestion as described in [30]. Only undigested products can be amplified and measured, allowing measurement of the difference between mock and B6-specifc ApaI-digested samples as an indicator of escape levels. At least two biological replicates were tested for each cloned line.

### Expression analysis using allelic RT-PCR

Total RNA including nascent RNA was prepared using the Qiagen QIAzol method and used for RT with random primers as well as no RT as the control of genomic DNA contamination. Samples from RT and no RT reactions were used for allelic PCR with B6- or *spretus*-specific primer pairs for *Car5b* intronic regions (P1, P2, and the enhancer; Additional file 1: Fig S7A, Additional file 2: Table S5) to evaluate escape levels of *Car5b* nascent transcripts in WT, Xi-Del1, Xi-Del2, and Xi-Inv1 lines.

## ATAC-seq, ChIP-seq, CUT&RUN, Hi-C, ChIP-PCR

ATAC-seq, CTCF ChIP-seq, and Hi-C datasets as well as allelic data analyses are described in our previous studies [16, 27, 43]. Virtual 4C analysis was performed using in situ DNase Hi-C data on Patski cells as described [27]. ChIP-seq for RAD21 was performed in WT and P4-edited Patski cells using a rabbit monoclonal antibody for RAD21 (Abcam ab217678) as described [16]. CUT&RUN was performed in WT and P4-edited Patski cells using antibodies for CTCF (Millipore 07–729), H3K27ac (Abcam ab4729) and H3K27me3 (Millipore 07–449), as described [43]. Allelic data analysis was done as described [27]. Additional CUT&RUN datasets of CTCF, H3K4me3, H3K27ac3, H3K36me3, and H3K27me3 in WT and *Firre* edited Patski cells were obtained from Thakur et al. [57]. Note that allelic analyses were not feasible in this batch due to limited SNP coverage in short (36nt) reads except for H3K4me3. ChIP was performed in WT, Xi-Del1, Xi-Del2, and Xi-Inv using antibodies for CTCF (Millipore 07–729), H3K27ac (Abcam ab4729) and RAD21 (Abcam ab992), followed by PCR using B6 Xi-specific primer pairs for P1, P2, the enhancer and P4 at the *Car5b* escape domain (Additional file 1: Fig S7A, Additional file 2: Table S5).

### CTCF motif analysis

DNA sequences (100-200 bp) of CTCF Xi-peaks in Patski cells were used to scan for conserved CTCF motifs, including detection of the Ren_20 motif and the MIT long motifs (LM2, LM7 and LM23), and determination of motif scores and orientation by CTCFBSDB 2.0 ([28]; Additional file 1: Table S1). Only CTCF Xi-peaks with a motif score ≥ 10 and clear orientation were considered to evaluate the CTCF binding pattern around the eight escapees chosen for this study. Note that for peaks containing two or more CTCF binding sites with a motif score ≥ 10, all motif sites are marked on the figures. Using a motif score > 3 cutoff, which is suggestive of a CTCF binding site, we obtained similar results.

### Xi-specific 3C-PCR analysis

To confirm the contacts between P4 and P1/P2 on the Xi in WT shown by virtual 4C analysis and test their occurrence in Xi-Inv, modified 3C chromatin DNA libraries were generated for WT, Xi-Del1, and Xi-Inv1 lines as previously described for the preparation of in situ Hi-C whole genome libraries with some modifications [58]. Two or three 3C experiments as biological replicates were done for each line. Briefly, for each 3C experiment, 1-2 M Patski cells were crosslinked with 1% formaldehyde at room temperature for 10 min, followed by glycine quenching, nuclei permeabilization and SDS lysis. Chromatin was digested with 800 units of DpnII, and the resulting chromatin fragments labeled using biotinylated dATP (Biotin-14-dATP, Cat #14,138, Active Motif), followed by in situ proximity ligation at 16 °C for 4 h. After reversal of crosslinks, the 3C DNA library was purified with Ampure beads (Agilent Court) and its concentration determined using a Nanodrop instrument. Note that enrichment by streptavidin pulldown, fragment sonication, and sequencing library preparation, used in Hi-C protocols were skipped. Aliquots of these 3C DNA libraries were checked for fragment length changes before and after proximity ligation to confirm successful ligation.

The 3C DNA libraries were diluted 1:4 to reach a concentration of ~ 10-15 ng/ul. 2ul was used for the 3C-PCR reaction with B6-specific primer pairs designed for 3C (Fig. 5A, Additional file 2: Table S5), which yielded products only from WT and Xi-Inv but not from Xi-Del (Fig. 5). Note that PCR products from chromatin contacts can vary in size between biological replicates, due to ligation of different DpnII sites from incomplete digestion. As a control, 20 ng genomic DNA from each of WT, Xi-Del1, and Xi-Inv1 lines was used for each PCR reaction using the same B6-specific primer pairs to confirm the lack of PCR products, which were only found in the 3C libraries (Fig. 5B, C).

## Supplementary Information

Additional file 1: Figure S1. Selection of eight escapees for domain and CTCF analysis. A, B. Escapees with a significant level of escape were selected based on allelic RNA-seq and ChIP-seq for RNA polymerase II in Patski cells (A) or brain (B) (Xi/Xi+Xa ratio ≥0.1 and expression ≥5 TPM/RPKM). Genes with low escape levels (highlighted in red) often do not have strong CTCF binding sites and thus were not included in escape domain analysis. By these criteria, no facultative escapees were chosen for brain. In addition to Car5b and Shroom4 additional four genes (Mid1^a^, Asmt^a^, Atp6ap1^b^ and 1810030O07Rik^c^) can be classified as facultative escapees in Patski cells. However, Mid1 spans the boundary of the PAR and Asmt is in the PAR, but the arrangement of these genes differs between B6 and Mus spretus, which complicates survey of CTCF binding patterns on the Xi and Xa [29], and led us to exclude these two genes for escape domain analysis. Atp6ap1 and 1810030O07Rik were also excluded as explained in C and D panels. C. RNA-seq reads for Atp6ap1 are located throughout the gene body on the Xa but are exclusively from the 5’ end of the gene on the Xi based on a single SNP, which suggests misassignment of reads due to an incorrect SNP. D. While 1810030O07Rik shows convincing evidence of escape, no CTCF binding was detected on the Xi or Xa at any regions within this gene or its neighbors subject to XCI. Figure S2. Escape domains in adult mouse brain. A. UCSC browser views of profiles of CTCF ChIP-seq reads on the Xa (blue) and Xi (green) are shown in domains around the facultative escapee Shroom4 and the constitutive escapees Kdm6a, Eif2s3x, Xist, Pbdc1, and Kdm5c in adult mouse brain. Putative escape domains are shaded in yellow, and silenced domains, in blue. CTCF peaks that flank escape domains around the constitutive escapees Kdm6a, Eif2s3x, Xist, Pbdc1, and Kdm5c are similar in Patski cells and mouse brain (see also the Ddx3x domain in Fig. 1B). In contrast, CTCF peaks around the facultative escapees Shroom4 (and Car5b, see Fig. 1C) present on the Xi in Patski cells but are absent in brain, consistent with silencing of these genes in brain. ChIP-seq data are from [16, 27]. Escapees are labeled in red, inactivated genes, in black, and genes with an unknown XCI status, in grey. See also Fig. 2A-F. B. Low levels of Gpr34 expression from the Xi are apparent in mouse adult brain, indicating escape from XCI. UCSC browser view of RNA-seq reads on the Xa (blue) and Xi (green) are shown at Gpr34. Figure S3. Allelic profiles of histone marks at escape domains in Patski cells. Profiles of H3K27ac as an active mark and of H3K27me3 as a repressive mark obtained by CUT&RUN on the Xa (blue) and Xi (green) at the escape domains of (A) the facultative escapee Shroom4, and (B-G) six constitutive escapees, Kdm6a, Ddx3x, Eif2s3x, Xist, Pbdc1, and Kdm5c. Escapees show enrichment in H3K27ac and depletion in H3K27me3 on the Xi, while neighbor genes subject to XCI show an opposite pattern. Putative escape domains are shaded in yellow, and silenced domains, in blue. See Fig. 4B-C for profiles at Car5b. ENCODE cCREs are shown for promoters (red), proximal and distal enhancers (yellow and orange), and conserved CTCF sites (cyan). The putative proximal/distal enhancer cCRE of an escapee is marked by an orange arrow based on the highest Xi-H3K27ac enrichment close to this escapee. Figure S4. Conserved expression of Car5b/CA5B in mouse and human tissues and escape status. A. Distribution of Car5b/CA5B expression in multiple tissues in mouse (blue) and human (red). Expression levels are shown in TPM from the Expression Atlas of EMBL-EBL for mouse and GTEX for human. B. Dynamic expression of Car5b during mouse development. Note that kidney shows the highest expression level of Car5b among tissues, which seems evident from embryonic stage ~E17. Expression levels are shown in TPM from the Expression Atlas of EMBL-EBL. C. Allelic expression analysis by B6-specific ApaI digestion shows that Car5b does not escape in the mouse adult tissues tested. PCR products from amplification of Car5b cDNA from mouse adult brain, spleen, heart, kidney, liver and ovary, and from MEFs, all with the Xi from spretus, and control genomic DNA (gDNA) from B6 and a F1 B6 x spretus heart sample were subject to mock or ApaI digestion (-/+) followed by gel electrophoresis. Complete digestion of PCR products from cDNA indicates the absence of transcripts from the Car5b allele on the spretus Xi, which lacks the ApaI site (also see additional file 1: Fig. S6A). D. Allelic Car5b expression levels measured by allelic read counts and TPMs in mouse tissues and Patski cells confirm that Car5b escapes in Patski cells but not in brain, spleen, and ovary based on the Xi/Xi+Xa expression ratio. RNA-seq data from [16, 27]. Figure S5. Allelic editing of CTCF site P4 at Car5b. A. Schematic of the ~2kb CTCF binding region P4 upstream of Car5b (Fig. 2B). The positions of the B6- and sp-specific sgRNA pairs used for CRISPR/Cas9 editing of the Xa (blue) and Xi (green) are marked by downward arrowheads. The positions of the non-allelic (F1, R1, F2, R2) and allelic primers (F, R; B6-R also used for allelic analysis of ChIP and 3C) are marked by black and colored arrows, respectively. The CTCF site P4 is marked by a red oval. B. Single-cell clones with deletion or inversion of the~2kb region on the Xi or Xa were selected and verified by PCR using combinations of primers flanking the cutting sites (downward arrows in A) followed by Sanger sequencing to verify allelic editing. Examples of analysis of Xi-Del2 and Xi-Inv1 clones are shown. The SNP positions and the junction sequences are indicated on the Sanger sequence. Note that the missing PCR products of F2/R2 from the sp Xa in Xi-edited lines (e.g., Xi-Del2 and Xi-Inv1) is because R2 carries 6nt that are only present in B6 (see Additional file 2: Table S5). In addition, PCR products of F1/R2 are 2126bp for the intact allele, which is often observed if increasing PCR extension time (middle panel). C. PCR with allelic primers (F, R) flanking region P4 further confirms correct editing events demonstrating loss of the B6-Xi-specific CTCF region P4 in Xi-Del1&2, with retention in Xi-Inv1 and Xa-Del1, while loss of the sp-Xa-specific CTCF region P4 occurred only in Xa-Del1, and not in Xi-Del1&2 and Xi-Inv1. Figure S6. Allelic expression analysis using B6-specific restriction enzyme digestion. A-B. B6-specific ApaI digestion shows that only Xi-deletion of the P4 site abolishes Car5b escape in Patski cells. A. (left) Cartoon to show B6-specific ApaI digestion which produces two bands with similar size since the Apa site is in the middle of B6 PCR products. (right) PCR products from genomic DNA from B6 mice or Patski cells were subject to mock or ApaI digestion (-/+) followed by gel electrophoresis, which confirms B6-specific ApaI digestion. B. PCR products from cDNA from P4-edited lines were subject to mock or ApaI digestion (-/+) followed by gel electrophoresis. Only lines with Xi-specific deletion of P4 (Xi-Del) show absence of the digested band, indicating loss of Car5b escape. Two biological replicates of each of the two deleted lines Xi-Del1&2 and one for each of the Xi-Inv lines were tested. Note that in one of the two replicates of Xi-Del2, a very weak digested band (red arrow) was observed, indicating a much lower level of Car5b escape compared to the control (Ctrl-a3). This is consistent with the results from Q-PCR analysis in this line (Fig. 3C). C-D. B6-specific BsrI digestion shows that editing of the CTCF region P4 located between Car5b and Siah1b on the Xi has no effects on Siah1b. C. PCR products from genomic DNA from B6 mice, cDNAs from F1 hybrid MEFs, and cDNA from Patski cells were subject to mock or ApaI digestion (-/+) followed by gel electrophoresis, which confirms B6-specific BsrI digestion and no escape of Siab1b in either WT MEFs or Patski cells. D. PCR products from cDNA from Del-Xi lines were subject to mock or BsrI digestion (-/+) followed by gel electrophoresis. The absence of digested bands indicates absence of Siab1b escape. Figure S7. Design and validation of allelic primer pairs at the Car5b domain. A. B6- or sp-specific primer pairs designed for P1, P2, the enhancer, and P4 are shown to complement Fig. 1A. The allelic primers for P4 were also used for confirmation of CTCF binding site editing (see Additional file 1: Fig. S5C). The sizes of amplicons are scaled and marked. Allelic primer pairs for P1, P2 and the enhancer are located in Car5b introns and thus can be used for analysis of nascent transcript levels (see Additional file 1: Fig. S8). B. Validation of allelic primer pairs using PCR with species-specific and Patski genomic DNA (gDNA). Primer pairs only yield PCR products from gDNA of their corresponding species. Figure S8. Escape levels of Car5b nascent transcripts are reduced after Xi-specific deletion but not inversion of P4. A. B6- or sp-specific primer pairs for intronic regions (P1, P2 and the enhancer) were used for PCR using samples treated with reverse transcriptase (RT) and controls (No RT). Nascent Car5b transcripts were detected both from the B6-Xi and sp-Xa in WT, Xi-Del1&2, and Xi-Inv1, with a reduction of Xi products in Xi-Del1&2, but not in Xi-Inv1. Lower levels were seen in Xi-Del1 than in Xi-Del2. No products were detected in the absence of RT, indicating no genomic DNA contamination. B. The amounts of PCR products were estimated by ImageJ to calculate Xi/Xi+Xa ratios of nascent Car5b transcripts. The mean ratio for the three regions measured (P1, P2 and enhancer) was plotted for WT (0.330, 0.333, 0.311), Xi-Del1 (0.079, 0.173, 0.018), Xi-Del2 (0.223, 0.268, 0.262) and Xi-Inv1 (0.331, 0.322, 0.359), and comparison between lines done by two-tail paired student’s t-test. Error bar: SEM. A significant decrease of escape levels of nascent transcripts is observed in Xi-Del1&2 lines, but not in Xi-Inv1 compared to WT, with the largest decrease in Xi-Del1, consistent with allelic expression analysis of exon products (see Fig. 3C and Additional file 1: Fig. S6B). Figure S9. Confirmation of CTCF contribution to protection of the Car5b escape domain from changes in histone modifications. Allelic profiles of CTCF (A), H3K27ac (B), and H3K27me3 (C) obtained by CUT&RUN are shown for the Car5b escape region in Xa-Del and in Xi-Del2. Similar but less pronounced effects than those observed in Xi-Del1 (Fig. 4A-C) are seen in Xi-Del2, including loss of CTCF peaks, loss of H3K27ac, and gain of H3K27me3 at Car5b. Figure S10. Xi-specific ChIP-PCR confirms that deletion but not inversion of P4 on the Xi affects CTCF, RAD21, and H3K27ac levels at P1, P2, and the enhancer in the Car5b escape domain. ChIP for CTCF, RAD21, and H3K27ac was performed in WT, Xi-Del1, Xi-Del2, and Xi-Inv1, followed by allelic PCR using B6-specific primer pairs targeting P1 (A, B), P2 (C, D), enhancer (E, F) and P4 (G, H). Gel images are shown in A, C, E, and G, and enrichment plots (density ratio between ChIP and input PCR products analyzed by ImageJ) in B, D, F, and H. A mock ChIP control (no antibody) is included. Primer design is shown in Additional file 1: Fig. S7. As expected, no Xi enrichment of any marks was detected at P4 in either of the two Xi-Del clones. The strongest enrichment in CTCF and RAD21 was detected at P4 compared to P1, P2, and the enhancer in both WT and Xi-Inv1. The second strongest CTCF enrichment was detected at P2, with a 10-20% decrease in Xi-Del clones but not in Xi-Inv1, compared to WT. Enrichment in CTCF at P1 and at the enhancer and enrichment of RAD21 at P1, P2, and the enhancer were too weak to evaluate changes between WT and edited clones. A strong enrichment in H3K27ac was observed at the enhancer, which shows a clear decrease in Xi-Del1 compared to WT, consistent with decrease Car5b escape in Xi-Del1. Figure S11. CTCF binding is required for enhancing Car5b escape in Patski cells with a Dxz4 deletion. A. Profiles of chromatin accessibility determined by ATAC-seq and of CTCF binding based on ChIP-seq are shown at Car5b on the Xa (blue) and Xi (green) in WT and Del-Hinge Patski cells. Only minor differences are observed between WT and De-Hinge profiles, consistent with a small increase of Car5b escape in Del-hinge (Fig. 6B). The CTCF sites P1-4 are marked, an orange box indicates the position of the enhancer and a red box that of the promoter. B. B6-specific ApaI digestion of RT-PCR products shows that Xi-specific deletion of P4 in Del-Hinge cells (Xi-Del3) strongly decreases Car5b escape (red arrow). In contrast, deletion of P4 on the Xa in Del-Hinge cells (Xa-Del2, Xa-Del3) shows no change compared to unedited Del-Hinge lines Dh-a7, Dh-a1. The gel electrophoresis image shows mock and ApaI digestion (-/+) of the PCR products. Figure S12. Allelic analysis of H3K4me3 in WT, Del-Firre, and Del-Firre+tg cells. Profiles of H3K4me3 enrichment determined by CUT&RUN on the Xa (blue) and Xi (green) are shown at Car5b in WT, Del-Firre, and Del-Firre+tg cells. H3K4me3 increases at the enhancer of Car5b in cells with Firre depletion (Del-Firre), consistent with increased expression from the Xi (see also Fig. 6E). This pattern is reversed in Del-Firre+tg cells. Figure S13. Inhibition of DNA methylation has no effect on Car5b escape but cause reactivation of neighbor genes subject to XCI. Allelic TPMs for the Xi allele, the Xa allele, and escape ratios (Xi/Xi+Xa expression) were plotted for Car5b and neighbor genes Ap1s2, Zrsf2, and Siah1b. RNA-seq data from [27]. Inhibition of DNA methylation was done by treatment of WT or Del-hinge Patski cells with 4 µM 5-aza-2′-deoxycytidine (aza), with effects compared to those in a DMSO mock control. Two biological replicates per treatment were done.

Additional file 2: Table S1. Information on motif analysis of CTCF Xi-peaks in Patski cells. Table S2. Information on escape domains on the X chromosome. Table S3. Information on allelic sgRNAs for CRISP/Cas9 editing and derived deletions and inversions. Table S4. Xi and Xa expression levels of eight escape genes in WT, Del-Firre and Del-Firre+tg. Table S5. Information on primers used

Additional file 3: Uncropped DNA gel images

## Data Availability

All data generated or analyzed during this study are included in this published article, its supplementary information files and publicly available repositories. Next-generation sequencing raw and processed data that support the findings of this study have been deposited in the National Centre for Biotechnology Information GEO and are accessible through the previously published GEO Series “GSE59779” [27, 43] and the Series “GSE231626” with NCBI BioProject Accession PRJNA967025 [59]. All other data, information and materials used for the analyses that support the findings of this study are available from the corresponding authors upon reasonable request.

## Abbreviations

3C: Chromosome conformation capture
4C: Circular chromosome conformation capture
BAC: Bacterial artificial chromosome
B6: C57B/6 J
cCRE: Candidate cis-regulatory element
CTCF: CCCTC-binding factor
Del: Deletion
Escapee: Escape gene
Hi-C: High-throughput genome-wide chromosome conformation capture
Inv: Inversion
Kb: Kilobase
MEF: Mouse embryonic fibroblast
PAR: Pseudoautosomal region
RT: Reverse transcription
SNP: Single nucleotide polymorphism
sp: Mus spretus
TAD: Topologically associated domain
TFs: Transcription factors
TPM/RPKM: Transcripts per million/reads per kilobase of transcript per million reads mapped
TSS: Transcription start site
WT: Wild type
XCI: X-chromosome inactivation
Xa: The active X chromosome
Xi: The inactive X chromosome
*Xist*: X-inactive specific transcript
