# Supplementary material for: CTCF-mediated insulation and chromatin environment modulate Car5b escape from X inactivation
Source: BMC Biol. 2025 Mar 3;23:68. doi: 10.1186/s12915-025-02137-7 (PMC11874400; doi:10.1186/s12915-025-02137-7)
Supplement: Supplementary file 1 — Additional file 1: Figure S1. Selection of eight escapees for domain and CTCF analysis. A, B. Escapees with a significant level of escape were selected based on allelic RNA-seq and ChIP-seq for RNA polymerase II in Patski cells (A) or brain (B) (Xi/Xi+Xa ratio ≥0.1 and expression ≥5 TPM/RPKM). Genes with low escape levels (highlighted in red) often do not have strong CTCF binding sites and thus were not included in escape domain analysis. By these criteria, no facultative escapees were chosen for brain. In addition to Car5b and Shroom4 additional four genes (Mid1a, Asmta, Atp6ap1b and 1810030O07Rikc) can be classified as facultative escapees in Patski cells. However, Mid1 spans the boundary of the PAR and Asmt is in the PAR, but the arrangement of these genes differs between B6 and Mus spretus, which complicates survey of CTCF binding patterns on the Xi and Xa [29], and led us to exclude these two genes for escape domain analysis. Atp6ap1 and 1810030O07Rik were also excluded as explained in C and D panels. C. RNA-seq reads for Atp6ap1 are located throughout the gene body on the Xa but are exclusively from the 5’ end of the gene on the Xi based on a single SNP, which suggests misassignment of reads due to an incorrect SNP. D. While 1810030O07Rik shows convincing evidence of escape, no CTCF binding was detected on the Xi or Xa at any regions within this gene or its neighbors subject to XCI. Figure S2. Escape domains in adult mouse brain. A. UCSC browser views of profiles of CTCF ChIP-seq reads on the Xa (blue) and Xi (green) are shown in domains around the facultative escapee Shroom4 and the constitutive escapees Kdm6a, Eif2s3x, Xist, Pbdc1, and Kdm5c in adult mouse brain. Putative escape domains are shaded in yellow, and silenced domains, in blue. CTCF peaks that flank escape domains around the constitutive escapees Kdm6a, Eif2s3x, Xist, Pbdc1, and Kdm5c are similar in Patski cells and mouse brain (see also the Ddx3x domain in Fig. 1B). In contrast, CTCF peaks around [file 12915_2025_2137_MOESM1_ESM.pdf]

Additional file 1:

Figure S1. Selection of eight escapees for domain and CTCF analysis. A, B. Escapees with a significant level of escape were selected based on allelic RNA-seq and ChIP-seq for RNA polymerase II in Patski cells (**A**) or brain (**B**) (Xi/Xi+Xa ratio ≥0.1 and expression ≥5 TPM/RPKM). Genes with low escape levels (highlighted in red) often do not have strong CTCF binding sites, and thus were not included in escape domain analysis. By these criteria, no facultative escapees were chosen for brain. In addition to *Car5b* and *Shroom4* additional four genes (*Mid1*[a], *Asmt*[a], *Atp6ap1*[b] and *1810030O07Rik*[c]) can be classified as facultative escapees in Patski cells. However, *Mid1* spans the boundary of the PAR and *Asmt* is in the PAR, but the arrangement of these genes differs between B6 and *Mus spretus*, which complicates survey of CTCF binding patterns on the Xi and Xa [29], and led us to exclude these two genes for escape domain analysis. *Atp6ap1* and *1810030O07Rik* were also excluded as explained in **C** and **D** panels. **C.** RNA-seq reads for *Atp6ap1* locate throughout the gene body on the Xa, but are exclusively from the 5' end of the gene on the Xi based on a single SNP, which suggests misassignment of reads due to an incorrect SNP. **D.** While *1810030O07Rik* shows convincing evidence of escape, no CTCF binding was detected on the Xi or Xa at any regions within this gene or its neighbors subject to XCI.

Figure S2. Escape domains in adult mouse brain. **A.** UCSC browser views of profiles of CTCF ChIP-seq reads on the Xa (blue) and Xi (green) are shown in domains around the facultative escapee *Shroom4* and the constitutive escapees *Kdm6a*, *Eif2s3x*, *Xist*, *Pbdc1*, and *Kdm5c* in adult mouse brain. Putative escape domains are shaded in yellow, and silenced domains, in blue. CTCF peaks that flank escape domains around the constitutive escapees *Kdm6a*, *Eif2s3x*, *Xist*, *Pbdc1*, and *Kdm5c* are similar in Patski cells and mouse brain (see also the *Ddx3x* domain in Fig. 1B). In contrast, CTCF peaks around the facultative escapees *Shroom4* (and *Car5b*, see Fig. 1C) present on the Xi in Patski cells but are absent in brain, consistent with silencing of these genes in brain. ChIP-seq data are from [16,27]. Escapees are labeled in red, inactivated genes, in black, and genes with an unknown XCI status, in grey. See also Fig. 2A-F. **B.** Low levels of *Gpr34* expression from the Xi are apparent in mouse adult brain, indicating escape from XCI. UCSC browser view of RNA-seq reads on the Xa (blue) and Xi (green) are shown at *Gpr34.*

Figure S3. Allelic profiles of histone marks at escape domains in Patski cells. Profiles of H3K27ac as an active mark and of H3K27me3 as a repressive mark obtained by CUT&RUN on the Xa (blue) and Xi (green) at the escape domains of (**A**) the facultative escapee *Shroom4*, and (**B-G**) six constitutive escapees, *Kdm6a*, *Ddx3x, Eif2s3x*, *Xist*, *Pbdc1*, and *Kdm5c*. Escapees show enrichment in H3K27ac and depletion in H3K27me3 on the Xi, while neighbor genes subject to XCI show an opposite pattern. Putative escape domains are shaded in yellow, and silenced domains, in blue. See Fig. 4B-C for profiles at *Car5b*. ENCODE cCREs are shown for promoters (red), proximal and distal enhancers (yellow and orange), and conserved CTCF sites (cyan). The putative proximal/distal enhancer cCRE of an escapee is marked by an orange arrow based on the highest Xi-H3K27ac enrichment close to this escapee.

Figure S4. Conserved expression of *Car5b/CA5B* in mouse and human tissues and escape status. **A.** Distribution of *Car5b/CA5B* expression in multiple tissues in mouse (blue) and human (red). Expression levels are shown in TPM from the Expression Atlas of EMBL-EBL for mouse and GTEX for human. **B.** Dynamic expression of *Car5b* during mouse development. Note that kidney shows the highest expression level of *Car5b* among tissues, which seems evident from embryonic stage ~E17. Expression levels are shown in TPM from the Expression Atlas of EMBL-EBL. **C.** Allelic expression analysis by B6-specific ApaI digestion shows that *Car5b* does not escape in the mouse adult tissues tested. PCR products from amplification of *Car5b* cDNA from mouse adult brain, spleen, heart, kidney, liver and ovary, and from MEFs, all with the Xi from *spretus*, and control genomic DNA (gDNA) from B6 and a F1 B6 x *spretus* heart sample were subject to mock or ApaI digestion (-/+) followed by gel electrophoresis. Complete digestion of PCR products from cDNA indicates the absence of transcripts from the *Car5b* allele on the *spretus* Xi, which lacks the ApaI site (also see additional file 1: Fig. S6A). **D.** Allelic *Car5b* expression levels measured by allelic read counts and TPMs in mouse tissues and Patski cells confirm that *Car5b* escapes in Patski cells but not in brain, spleen, and ovary based on the Xi/Xi+Xa expression ratio. RNA-seq data from [16,27].

Figure S5. Allelic editing of CTCF site P4 at *Car5b*. A. Schematic of the ~2kb CTCF binding region P4 upstream of *Car5b* (Fig. 2B). The positions of the B6- and *sp*-specific sgRNA pairs used for CRISPR/Cas9 editing of the Xa (blue) and Xi (green) are marked by downward arrowheads. The positions of the non-allelic (F1, R1, F2, R2) and allelic primers (F, R; B6-R also used for allelic analysis of ChIP and 3C) are marked by black and colored arrows, respectively. The CTCF site P4 is marked by a red oval. **B.** Single-cell clones with deletion or inversion of the ~2kb region on the Xi or Xa were selected and verified by PCR using combinations of primers flanking the cutting sites (downward arrows in A) followed by Sanger sequencing to verify allelic editing. Examples of analysis of Xi-Del2 and Xi-Inv1 clones are shown. The SNP positions and the junction sequences are indicated on the Sanger sequence. Note that the missing PCR products of F2/R2 from the sp Xa in Xi-edited lines (e.g., Xi-Del2 and Xi-Inv1) is because R2 carries 6nt that are only present in B6 (see Additional file 2: Table S5). In addition, PCR products of F1/R2 are 2126bp for the intact allele, which is often observed if increasing PCR extension time (middle panel). **C.** PCR with allelic primers (F, R) flanking region P4 further confirms correct editing events demonstrating loss of the B6-Xi-specific CTCF region P4 in Xi-Del1&2, with retention in Xi-Inv1 and Xa-Del1, while loss of the *sp*-Xa-specific CTCF region P4 occurred only in Xa-Del1, and not in Xi-Del1&2 and Xi-Inv1.

Figure S6. Allelic expression analysis using B6-specific restriction enzyme digestion. A-B. B6-specific ApaI digestion shows that only Xi-deletion of the P4 site abolishes *Car5b* escape in Patski cells. **A.** (left) Cartoon to show B6-specific ApaI digestion which produces two bands with similar size since the Apa site is in the middle of B6 PCR products. (right) PCR products from genomic DNA from B6 mice or Patski cells were subject to mock or ApaI digestion (-/+) followed by gel electrophoresis, which confirms B6-specific ApaI digestion. **B.** PCR products from cDNA from P4-edited lines were subject to mock or ApaI digestion (-/+) followed by gel electrophoresis. Only lines with Xi-specific deletion of P4 (Xi-Del) show absence of the digested band, indicating loss of *Car5b* escape. Two biological replicates of each of the two deleted lines Xi-Del1&2 and one for each of

the Xi-Inv lines were tested. Note that in one of the two replicates of Xi-Del2, a very weak digested band (red arrow) was observed, indicating a much lower level of *Car5b* escape compared to the control (Ctrl-a3). This is consistent with the results from Q-PCR analysis in this line (Fig. 3C). **C-D**. B6-specific BsrI digestion shows that editing of the CTCF region P4 located between *Car5b* and *Siah1b* on the Xi has no effects on *Siah1b*. **C.** PCR products from genomic DNA from B6 mice, cDNAs from F1 hybrid MEFs, and cDNA from Patski cells were subject to mock or ApaI digestion (-/+) followed by gel electrophoresis, which confirms B6-specific BsrI digestion and no escape of *Siab1b* in either WT MEFs or Patski cells. **D.** PCR products from cDNA from Del-Xi lines were subject to mock or BsrI digestion (-/+) followed by gel electrophoresis. The absence of digested bands indicates absence of *Siab1b* escape.

Figure S7. Design and validation of allelic primer pairs at the *Car5b* domain. A. B6- or sp-specific primer pairs designed for P1, P2, the enhancer, and P4 are shown to complement Fig. 1A. The allelic primers for P4 were also used for confirmation of CTCF binding site editing (see Additional file 1: Fig. S5C). The sizes of amplicons are scaled and marked. Allelic primer pairs for P1, P2 and the enhancer are located in *Car5b* introns and thus can be used for analysis of nascent transcript levels (see Additional file 1: Fig. S8). **B.** Validation of allelic primer pairs using PCR with species-specific and Patski genomic DNA (gDNA). Primer pairs only yield PCR products from gDNA of their corresponding species.

Figure S8. Escape levels of *Car5b* nascent transcripts are reduced after Xi-specific deletion but not inversion of P4. A. B6- or sp-specific primer pairs for intronic regions (P1, P2 and the enhancer) were used for PCR using samples treated with reverse transcriptase (RT) and controls (No RT). Nascent *Car5b* transcripts were detected both from the B6-Xi and sp-Xa in WT, Xi-Del1&2, and Xi-Inv1, with a reduction of Xi products in Xi-Del1&2, but not in Xi-Inv1. Lower levels were seen in Xi-Del1 than in Xi-Del2. No products were detected in the absence of RT, indicating no genomic DNA contamination. **B.** The amounts of PCR products were estimated by ImageJ to calculate Xi/Xi+Xa ratios of nascent *Car5b* transcripts. The mean ratio for the three regions measured (P1, P2 and enhancer) was plotted for WT (0.330, 0.333, 0.311), Xi-Del1 (0.079, 0.173, 0.018), Xi-Del2 (0.223, 0.268, 0.262) and Xi-Inv1 (0.331, 0.322, 0.359), and comparison between lines done by two-tail paired student's t-test. Error bar: SEM. A significant decrease of escape levels of nascent transcripts is observed in Xi-Del1&2 lines, but not in Xi-Inv1 compared to WT, with the largest decrease in Xi-Del1, consistent with allelic expression analysis of exon products (see Fig. 3C and Additional file 1: Fig. S6B).

Figure S9. Confirmation of CTCF contribution to protection of the *Car5b* escape domain from changes in histone modifications. Allelic profiles of CTCF (**A**), H3K27ac (**B**), and H3K27me3 (**C**) obtained by CUT&RUN are shown for the *Car5b* escape region in Xa-Del and in Xi-Del2. Similar but less pronounced effects than those observed in Xi-Del1 (Fig. 4A-C) are seen in Xi-Del2, including loss of CTCF peaks, loss of H3K27ac, and gain of H3K27me3 at *Car5b*.

Figure S10. Xi-specific ChIP-PCR confirms that deletion but not inversion of P4 on the Xi affects CTCF, RAD21, and H3K27ac levels at P1, P2, and the enhancer in the *Car5b* escape domain. ChIP for CTCF, RAD21, and H3K27ac was performed in WT, Xi-Del1, Xi-Del2, and Xi-Inv1, followed by allelic PCR using B6-

specific primer pairs targeting P1 (**A, B**), P2 (**C, D**), enhancer (**E, F**) and P4 (**G, H**). Gel images are shown in **A, C, E,** and **G,** and enrichment plots (density ratio between ChIP and input PCR products analyzed by ImageJ) in **B, D, F**, and **H**. A mock ChIP control (no antibody) is included. Primer design is shown in Additional file 1: Fig. S7. As expected, no Xi enrichment of any marks was detected at P4 in either of the two Xi-Del clones. The strongest enrichment in CTCF and RAD21 was detected at P4 compared to P1, P2, and the enhancer in both WT and Xi-Inv1. The second strongest CTCF enrichment was detected at P2, with a 10-20% decrease in Xi-Del clones but not in Xi-Inv1, compared to WT. Enrichment in CTCF at P1 and at the enhancer and enrichment of RAD21 at P1, P2, and the enhancer were too weak to evaluate changes between WT and edited clones. A strong enrichment in H3K27ac was observed at the enhancer, which shows a clear decrease in Xi-Del1 compared to WT, consistent with decrease *Car5b* escape in Xi-Del1.

Figure S11. CTCF binding is required for enhancing *Car5b* escape in Patski cells with a *Dxz4* deletion. A. Profiles of chromatin accessibility determined by ATAC-seq and of CTCF binding based on ChIP-seq are shown at *Car5b* on the Xa (blue) and Xi (green) in WT and Del-Hinge Patski cells. Only minor differences are observed between WT and De-Hinge profiles, consistent with a small increase of *Car5b* escape in Del-hinge (Fig. 6B). The CTCF sites P1-4 are marked, an orange box indicates the position of the enhancer and a red box that of the promoter. **B.** B6-specific ApaI digestion of RT-PCR products shows that Xi-specific deletion of P4 in Del-Hinge cells (Xi-Del3) strongly decreases *Car5b* escape (red arrow). In contrast, deletion of P4 on the Xa in Del-Hinge cells (Xa-Del2, Xa-Del3) shows no change compared to unedited Del-Hinge lines Dh-a7, Dh-a1. The gel electrophoresis image shows mock and ApaI digestion (-/+) of the PCR products.

Figure S12. Allelic analysis of H3K4me3 in WT, Del-Firre, and Del-Firre+tg cells. Profiles of H3K4me3 enrichment determined by CUT&RUN on the Xa (blue) and Xi (green) are shown at *Car5b* in WT, Del-Firre, and Del-Firre+tg cells. H3K4me3 increases at the enhancer of *Car5b* in cells with *Firre* depletion (Del-Firre), consistent with increased expression from the Xi (see also Fig. 6E). This pattern is reversed in Del-Firre+tg cells.

Figure S13. Inhibition of DNA methylation has no effect on *Car5b* escape but cause reactivation of neighbor genes subject to XCI. Allelic TPMs for the Xi allele, the Xa allele, and escape ratios (Xi/Xi+Xa expression) were plotted for *Car5b* and neighbor genes *Ap1s2*, *Zrsf2*, and *Siah1b*. RNA-seq data from [27]. Inhibition of DNA methylation was done by treatment of WT or Del-hinge Patski cells with 4 μM 5-aza-2′-deoxycytidine (aza), with effects compared to those in a DMSO mock control. Two biological replicates per treatment were done.

Fig. S1

A

Escape genes_Patski	Escape ratio Xi/(Xi+Xa)	TPM	TSS-PolII Xi read	TSS-PolII Xa read	TSS-PolII Xi/(Xa+Xi)
Xist	0.999	102.6	9.4	0.4	0.96
Pbdc1	0.419	35.7	12.3	14	0.47
Kdm6a	0.337	36.4	8	8.7	0.48
Eif2s3x	0.333	172.6	8.9	20.5	0.30
Shroom4	0.320	38.4	5.6	21.5	0.21
Kdm5c	0.291	46.9	4.6	7.8	0.37
Ddx3x	0.228	289.3	118.9	167.2	0.42
Car5b	0.173	34.9	1	3.6	0.22
Mid1 [a]	0.241	54.5	1.6	3.8	0.30
Asmt [a]	0.308	10.1	na	na	na
Atp6ap1 [b]	0.268	330.0	26.2	24	0.52
1810030O07Rik [c]	0.259	55.6	12.6	7.5	0.63
Zfp300	0.700	2.1	3	5.8	0.34
Ftx	0.397	1.1	1.5	1.3	0.54
5530601H04Rik	0.330	4.9	7	10.6	0.40
Xkrx	0.300	3.6	2.4	4.8	0.33
Gm14827	0.246	1.4	2.4	4.9	0.33
Slc16a2	0.162	2.0	0.5	2.3	0.18
Tmem29	0.116	16.7	0	3.1	0.00
Rp2h	0.308	17.8	1	11.6	0.08
Efnb1	0.054	138.1	28.5	47.1	0.38
Pim2	0.049	17.0	0.6	15.3	0.04
Magt1	0.044	64.9	1.7	7.6	0.18
Hmgn5	0.031	40.0	0.5	4.6	0.10
Yipf6	0.023	21.7	4.4	15.4	0.22
Gnl3l	0.022	63.3	1.6	11.5	0.12
Sh3bgrl	0.018	162.4	0.2	2.2	0.08
Rlim	0.017	51.0	23.7	23.8	0.50

B

Escape genes_brain	Escape ratio Xi/(Xi+Xa)	RPKM	TSS-PolII Xi read	TSS-PolII Xa read	TSS-PolII Xi/(Xa+Xi)
Xist	0.998	60.9	6	0.5	0.92
Kdm6a	0.264	10.3	7.7	17.6	0.30
Eif2s3x	0.252	71.3	15.3	24.6	0.38
Kdm5c	0.232	7.0	8.1	31.8	0.20
Ddx3x	0.225	88.9	142	252.9	0.36
Pbdc1	0.091	7.4	9.3	26.6	0.26
5530601H04Rik	0.203	3.8	11.6	23.5	0.33
Slc16a2	0.085	5.1	0.5	16.1	0.03
Ftx	0.078	14.0	6.2	25.4	0.20
Tmem47	0.013	52.2	0.3	9.6	0.03
Gpm6b	0.012	355.9	0.3	7.9	0.04
Gdi1	0.009	123.7	0.8	48.9	0.02
Plp1	0.003	902.8	1.4	12.7	0.10
Syp	0.003	279.5	0.6	22.3	0.03
Gprasp1	0.003	99.8	0.9	37.2	0.02

C

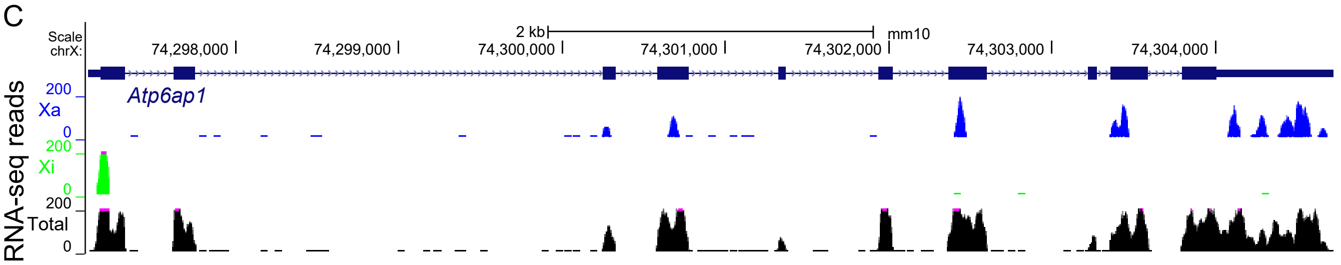

D

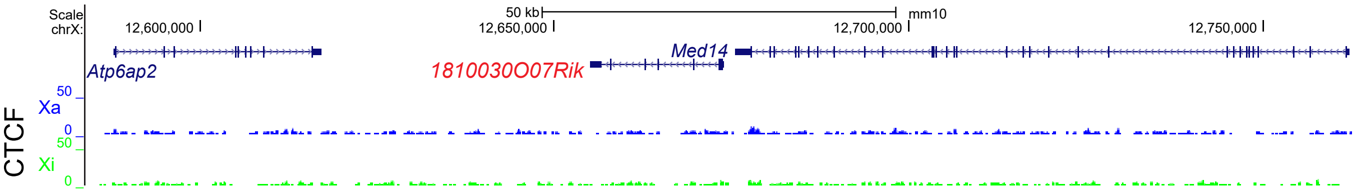

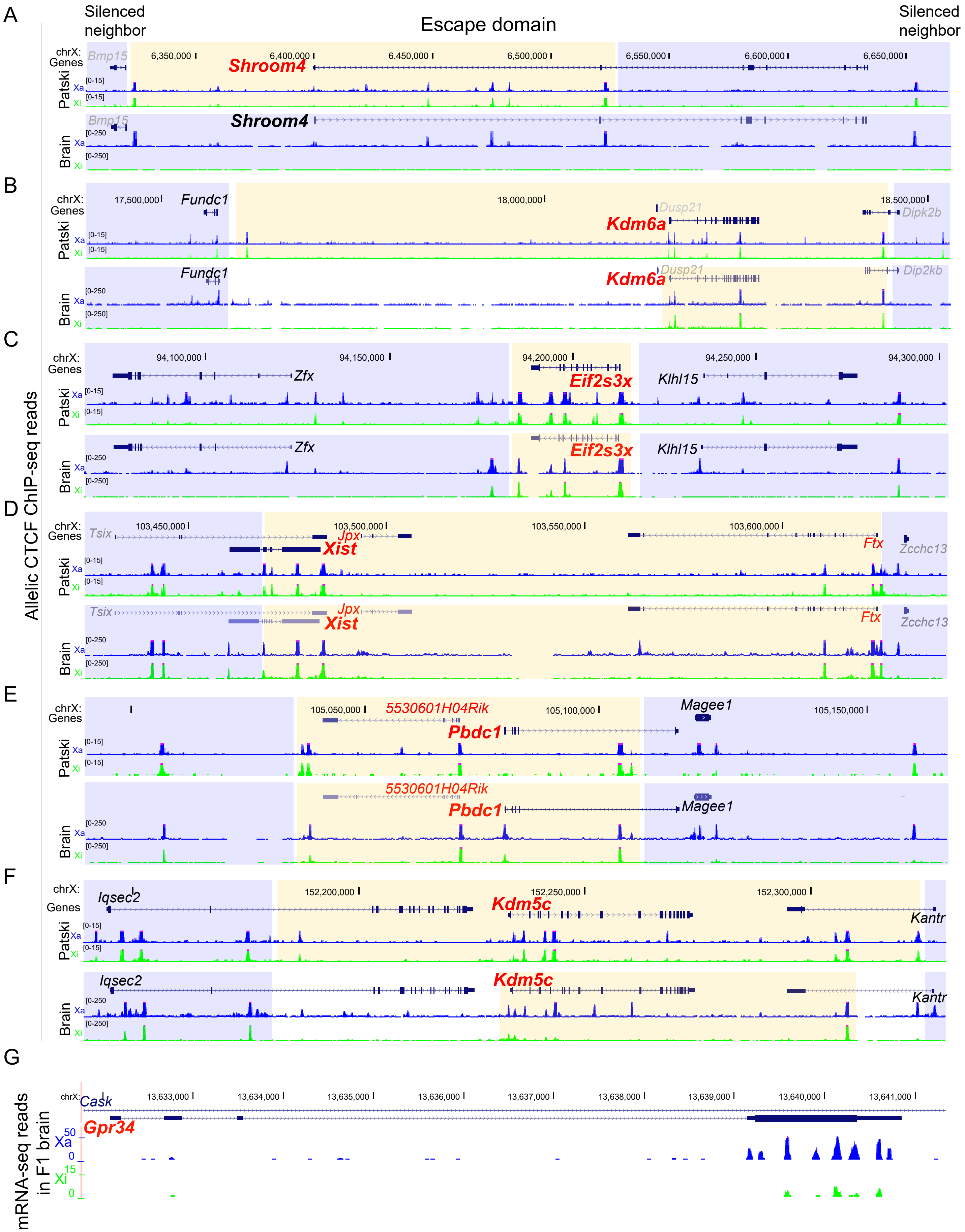
A
Silenced neighbor
Escape domain
Silenced neighbor
chrX:
Genes
Bmp15
Shroom4
Patski
Brain
Xa
Xi
B
Fundc1
Kdm6a
Dusp21
Dipk2b
C
Zfx
Eif2s3x
Klhl15
D
Tsix
Jpx
Xist
Ftx
Zcchc13
E
5530601H04Rik
Pbdc1
Magee1
F
Iqsec2
Kdm5c
Kantr
G
Cask
Gpr34
Allelic CTCF ChIP-seq reads
mRNA-seq reads in F1 brain

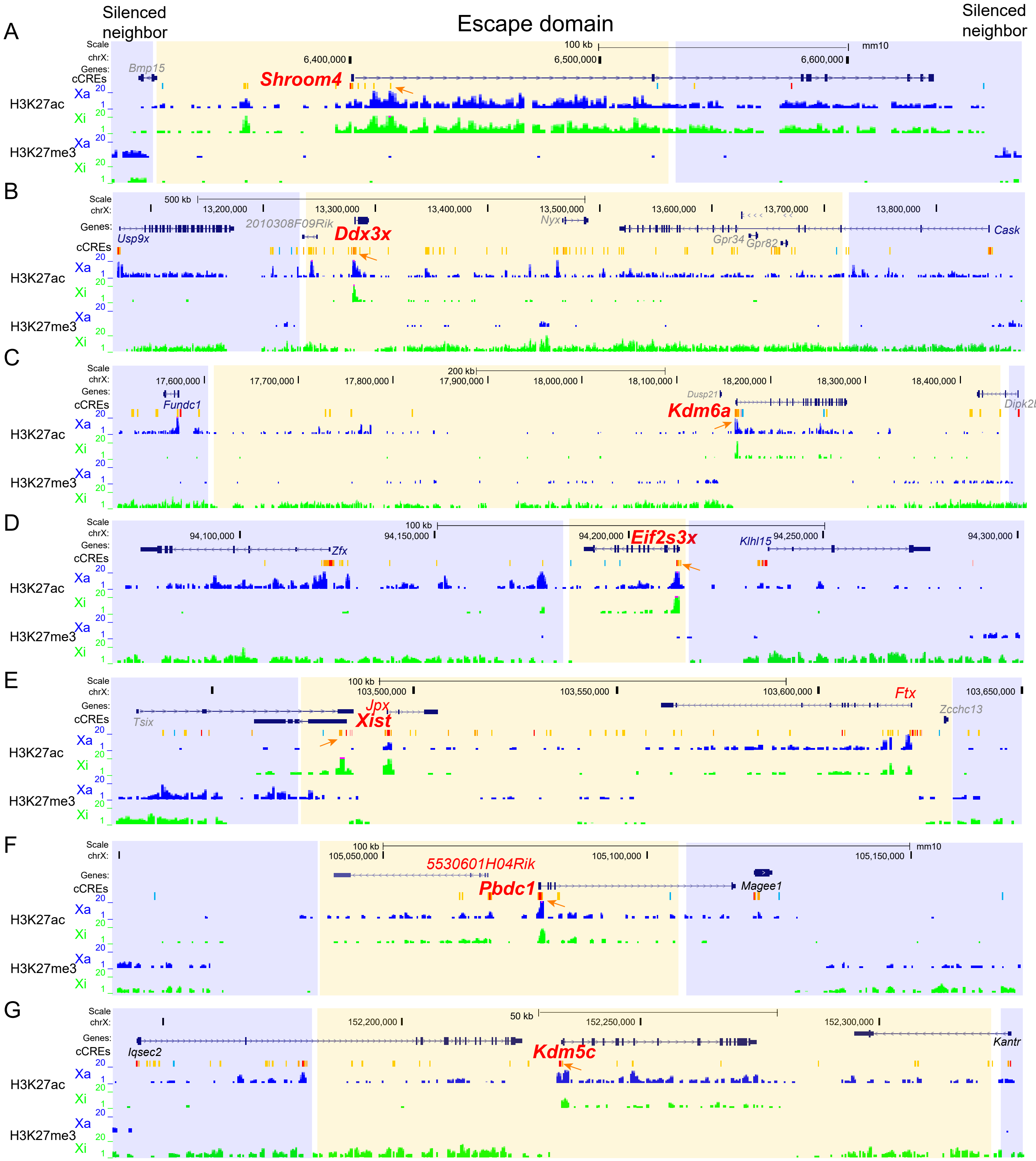
A
Silenced neighbor
Escape domain
Silenced neighbor
Scale
100 kb
mm10
chrX:
6,400,000
6,500,000
6,600,000
Genes:
Bmp15
Shroom4
cCREs
H3K27ac
Xa
Xi
H3K27me3
Xa
Xi
B
500 kb
13,200,000
13,300,000
13,400,000
13,500,000
13,600,000
13,700,000
13,800,000
Usp9x
2010308F09Rik
Ddx3x
Nyx
Gpr34
Gpr82
Cask
C
200 kb
17,600,000
17,700,000
17,800,000
17,900,000
18,000,000
18,100,000
18,200,000
18,300,000
18,400,000
Fundc1
Dusp21
Kdm6a
Dipk2b
D
100 kb
94,100,000
94,150,000
94,200,000
94,250,000
94,300,000
Zfx
Eif2s3x
Klhl15
E
100 kb
103,500,000
103,550,000
103,600,000
103,650,000
Tsix
Jpx
Xist
Ftx
Zcchc13
F
100 kb
mm10
105,050,000
105,100,000
105,150,000
5530601H04Rik
Pbdc1
Magee1
G
50 kb
152,200,000
152,250,000
152,300,000
Iqsec2
Kdm5c
Kantr

Fig. S4

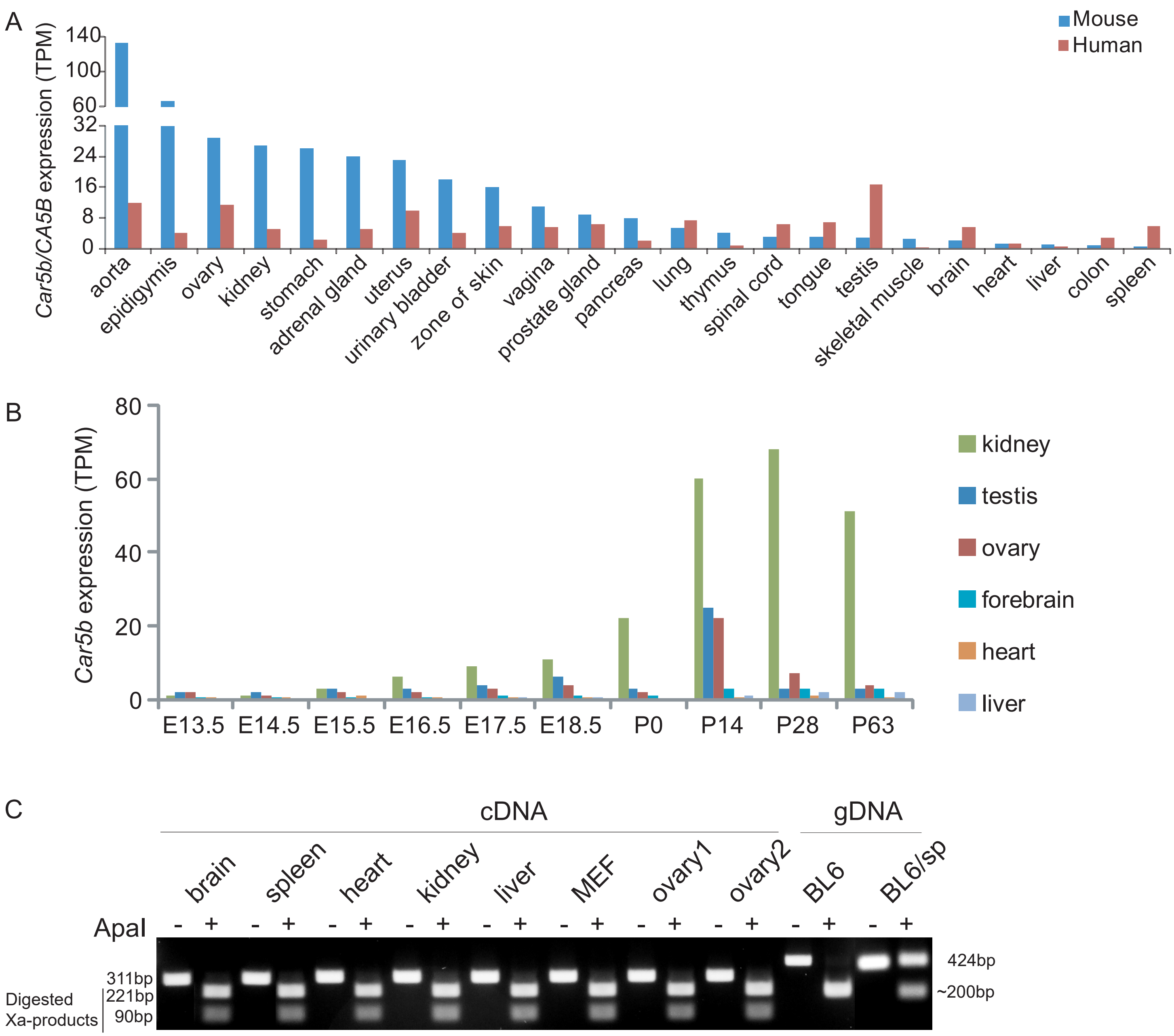

D

Car5b	Mouse adult tissues					Cell line_E18.5 kidney	
	brain1	brain2	spleen1	spleen2	ovary	Patski-rep1	Patski-rep2
TPM	1.2	1.4	0.5	0.2	23.3	37.8	35.2
Xa read counts	107	27	75	9	2290	4396	4302
Xi reads counts	3	0	1	0	21	1237	1162
Xa-TPM	1.1	1.4	0.5	0.2	23.1	29.5	27.7
Xi-TPM	0.03	0	0.01	0	0.21	8.3	7.5
Xi/total ratio	0.03	0	0.01	0	0.01	0.22	0.21

Fig. S5

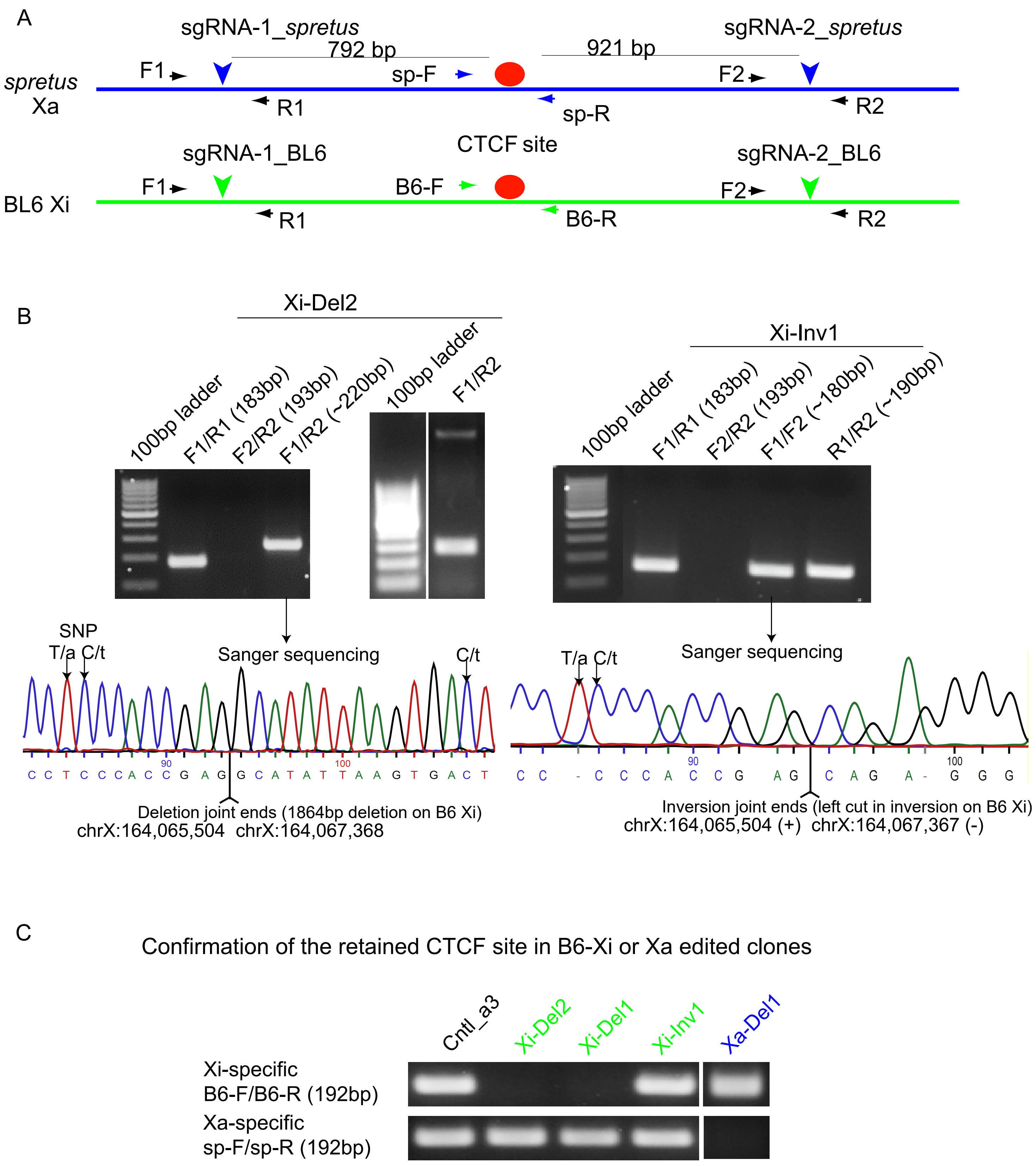

A
sgRNA-1_spretus
792 bp
921 bp
sgRNA-2_spretus
spretus
Xa
F1
R1
sp-F
sp-R
F2
R2
CTCF site
sgRNA-1_BL6
sgRNA-2_BL6
BL6 Xi
F1
R1
B6-F
B6-R
F2
R2
B
Xi-Del2
100bp ladder
F1/R1 (183bp)
F2/R2 (193bp)
F1/R2 (~220bp)
100bp ladder
F1/R2
Xi-Inv1
100bp ladder
F1/R1 (183bp)
F2/R2 (193bp)
F1/F2 (~180bp)
R1/R2 (~190bp)
SNP
T/a C/t
Sanger sequencing
C/t
C C T C C C A C C G A G G C A T A T T A A G T G A C T
Deletion joint ends (1864bp deletion on B6 Xi)
chrX:164,065,504 chrX:164,067,368
T/a C/t
Sanger sequencing
C C - C C C A C C G A G C A G A - G G G
Inversion joint ends (left cut in inversion on B6 Xi)
chrX:164,065,504 (+) chrX:164,067,367 (-)
C
Confirmation of the retained CTCF site in B6-Xi or Xa edited clones
Cntl_a3
Xi-Del2
Xi-Del1
Xi-Inv1
Xa-Del1
Xi-specific
B6-F/B6-R (192bp)
Xa-specific
sp-F/sp-R (192bp)

Fig. S6

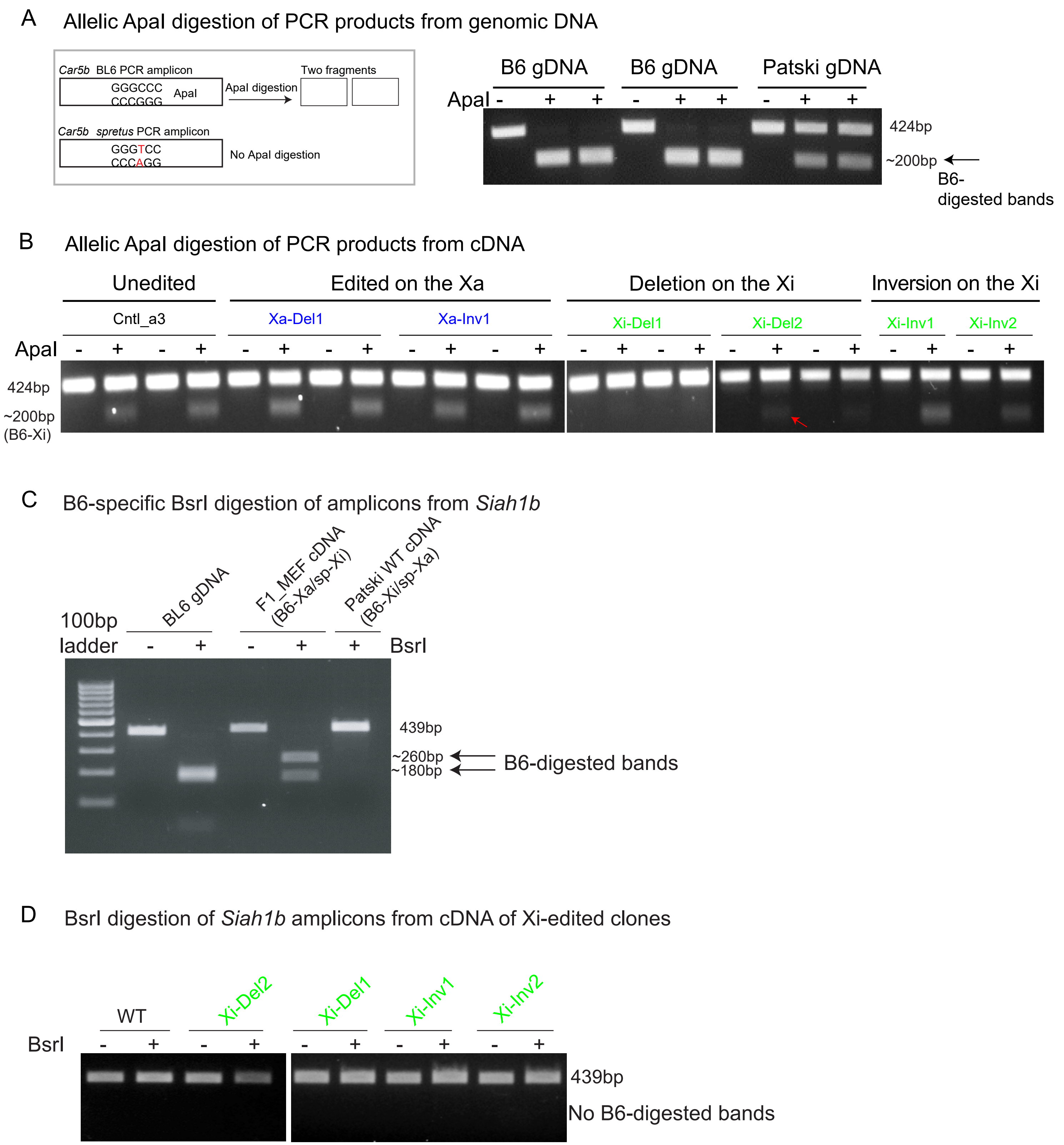

A
Allelic ApaI digestion of PCR products from genomic DNA
Car5b BL6 PCR amplicon
GGGCCC
CCCGGG
ApaI
ApaI digestion
Two fragments
Car5b spretus PCR amplicon
GGGTCC
CCCAGG
No ApaI digestion
B6 gDNA
B6 gDNA
Patski gDNA
ApaI
424bp
~200bp
B6-digested bands
B
Allelic ApaI digestion of PCR products from cDNA
Unedited
Edited on the Xa
Deletion on the Xi
Inversion on the Xi
Cntl_a3
Xa-Del1
Xa-Inv1
Xi-Del1
Xi-Del2
Xi-Inv1
Xi-Inv2
ApaI
424bp
~200bp (B6-Xi)
C
B6-specific BsrI digestion of amplicons from Siah1b
100bp ladder
BL6 gDNA
F1_MEF cDNA (B6-Xa/sp-Xi)
Patski WT cDNA (B6-Xi/sp-Xa)
BsrI
439bp
~260bp
~180bp
B6-digested bands
D
BsrI digestion of Siah1b amplicons from cDNA of Xi-edited clones
WT
Xi-Del2
Xi-Del1
Xi-Inv1
Xi-Inv2
BsrI
439bp
No B6-digested bands

Fig S7

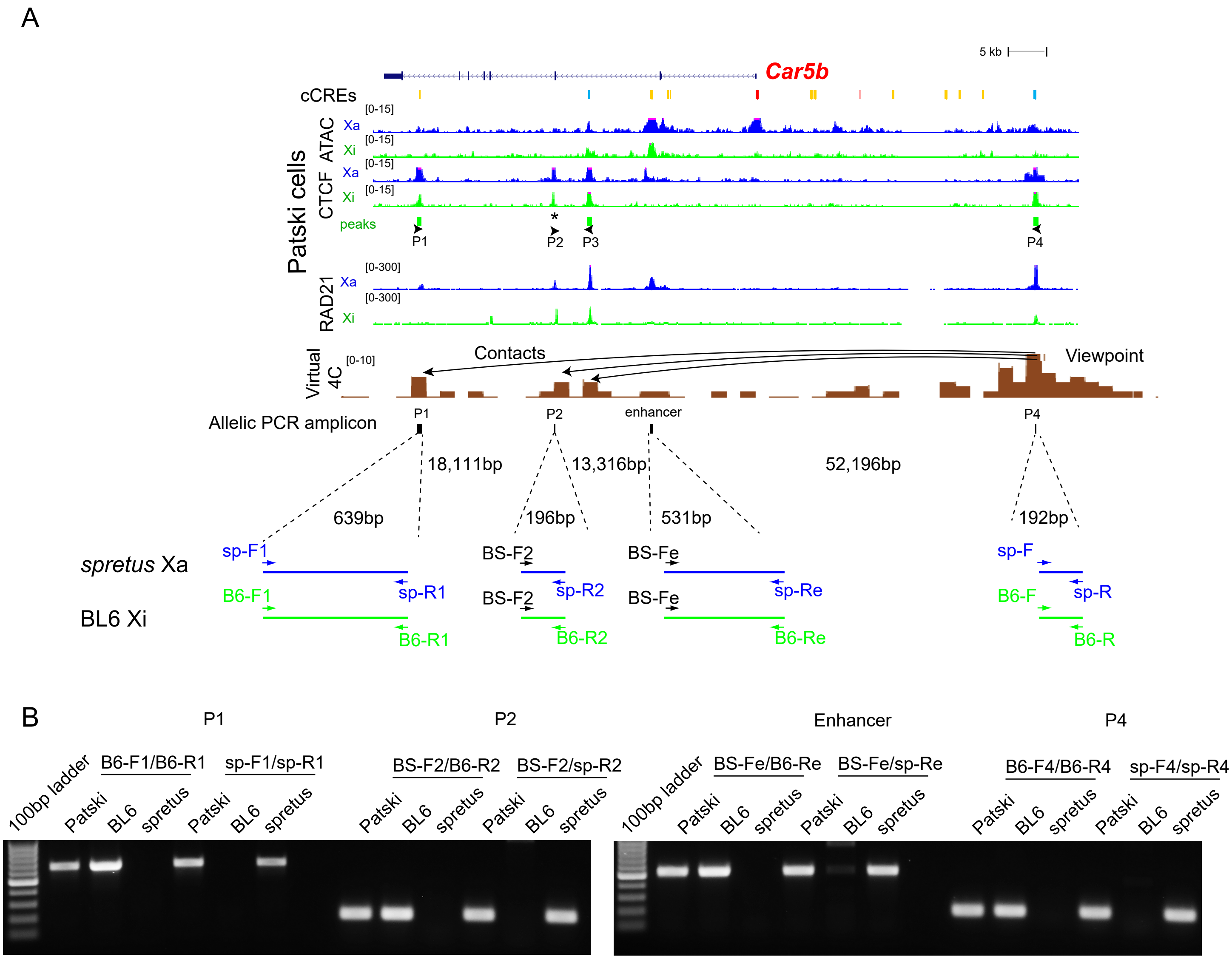
A
5 kb
Car5b
cCREs
Patski cells
ATAC
Xa
Xi
CTCF
Xa
Xi
peaks
P1
P2
P3
P4
RAD21
Xa
Xi
[0-15]
[0-300]
Virtual 4C
[0-10]
Contacts
Viewpoint
Allelic PCR amplicon
P1
P2
enhancer
P4
18,111bp
13,316bp
52,196bp
639bp
196bp
531bp
192bp
spretus Xa
BL6 Xi
sp-F1
sp-R1
B6-F1
B6-R1
BS-F2
sp-R2
B6-R2
BS-Fe
sp-Re
B6-Re
sp-F
sp-R
B6-F
B6-R
B
P1
P2
Enhancer
P4
100bp ladder
B6-F1/B6-R1
sp-F1/sp-R1
BS-F2/B6-R2
BS-F2/sp-R2
BS-Fe/B6-Re
BS-Fe/sp-Re
B6-F4/B6-R4
sp-F4/sp-R4
Patski
BL6
spretus

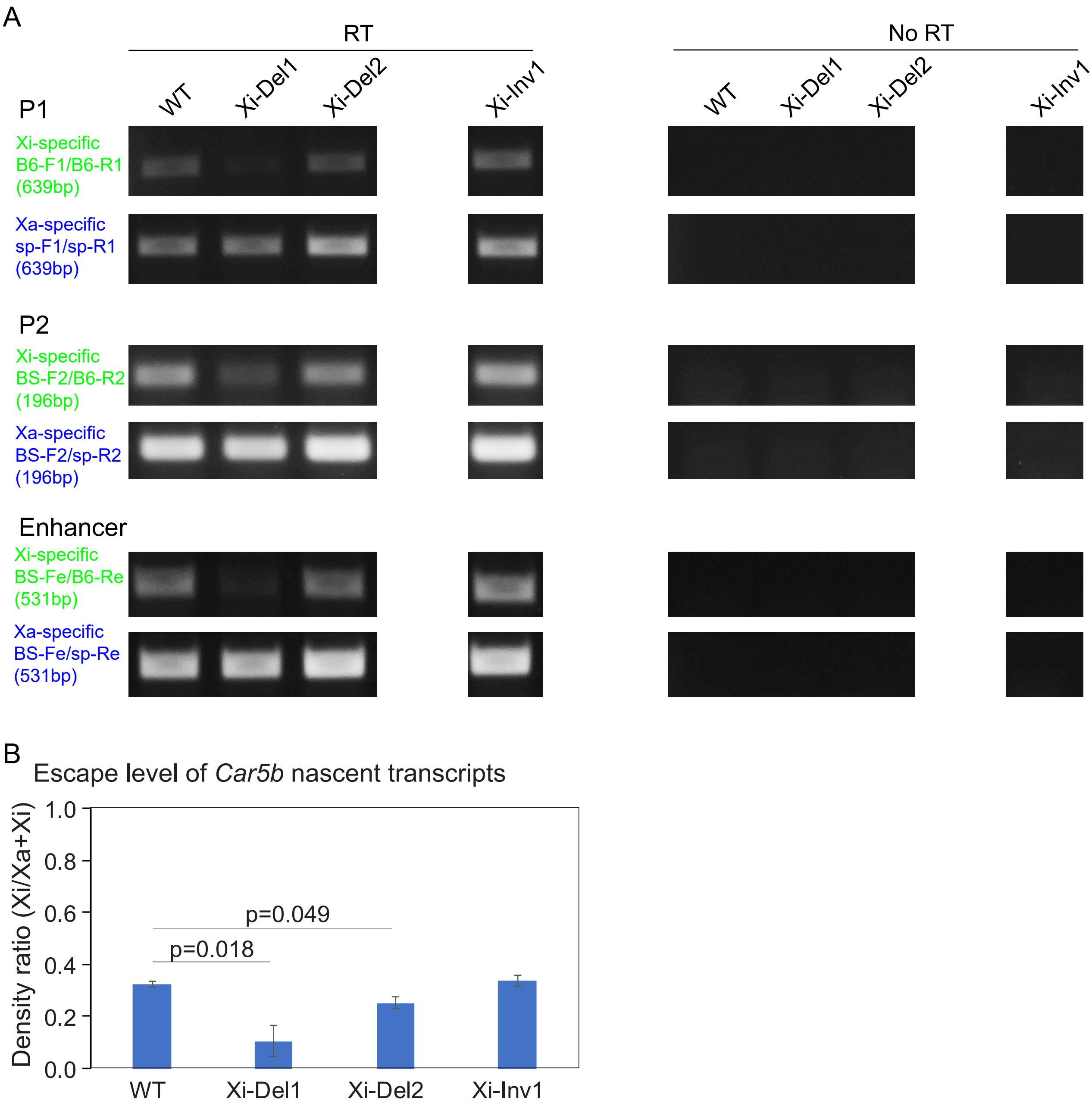
A
RT
No RT
WT
Xi-Del1
Xi-Del2
Xi-Inv1
P1
Xi-specific
B6-F1/B6-R1
(639bp)
Xa-specific
sp-F1/sp-R1
(639bp)
P2
Xi-specific
BS-F2/B6-R2
(196bp)
Xa-specific
BS-F2/sp-R2
(196bp)
Enhancer
Xi-specific
BS-Fe/B6-Re
(531bp)
Xa-specific
BS-Fe/sp-Re
(531bp)
B
Escape level of Car5b nascent transcripts
Density ratio (Xi/Xa+Xi)
1.0
0.8
0.6
0.4
0.2
0.0
p=0.049
p=0.018
WT
Xi-Del1
Xi-Del2
Xi-Inv1

Fig. S9

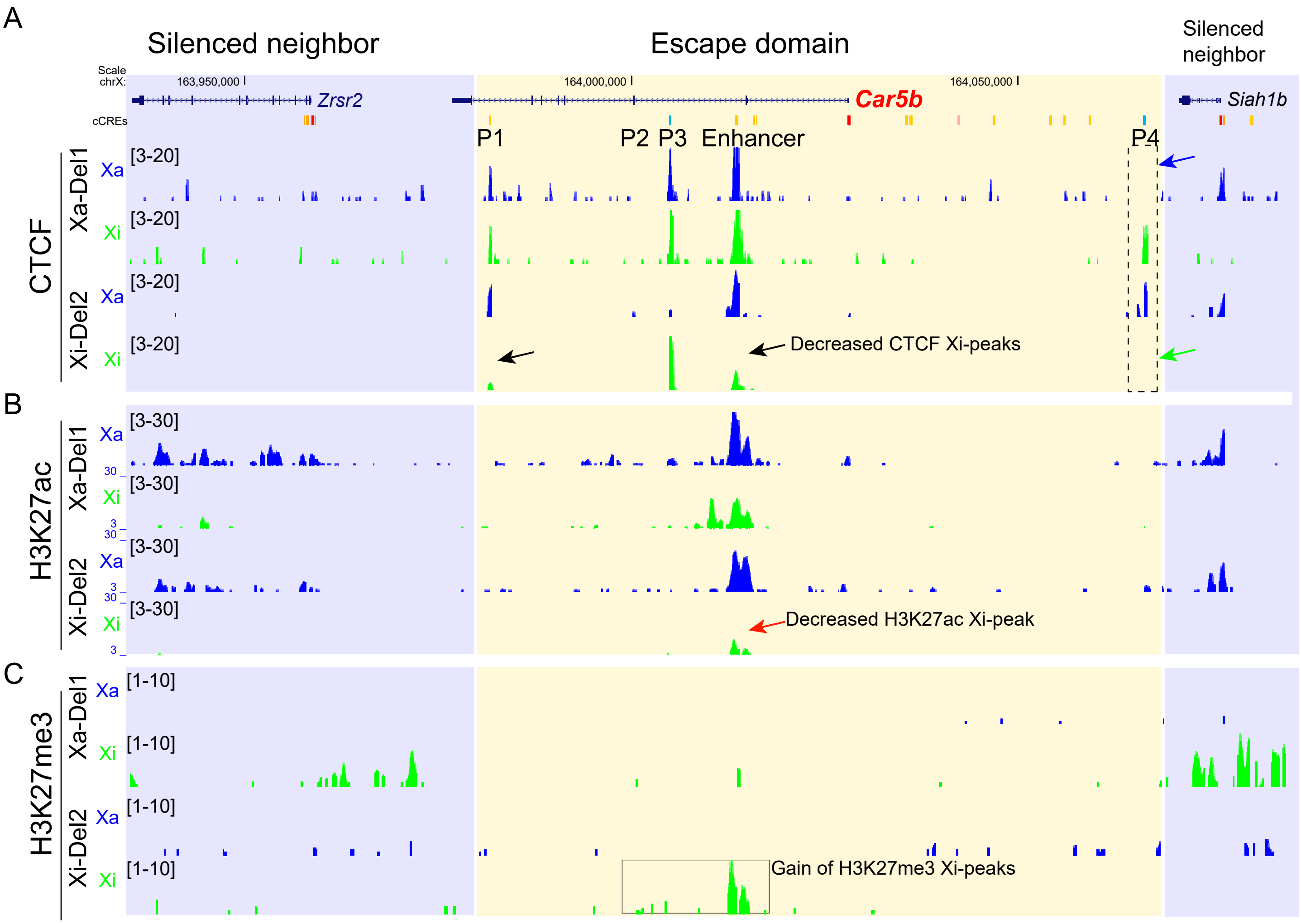
A
Silenced neighbor
Escape domain
Silenced neighbor
Scale
chrX:
163,950,000
164,000,000
164,050,000
Zrsr2
Car5b
Siah1b
cCREs
P1
P2
P3
Enhancer
P4
CTCF
Xa-Del1
Xi-Del2
Xa
Xi
[3-20]
Decreased CTCF Xi-peaks
B
H3K27ac
[3-30]
Decreased H3K27ac Xi-peak
C
H3K27me3
[1-10]
Gain of H3K27me3 Xi-peaks

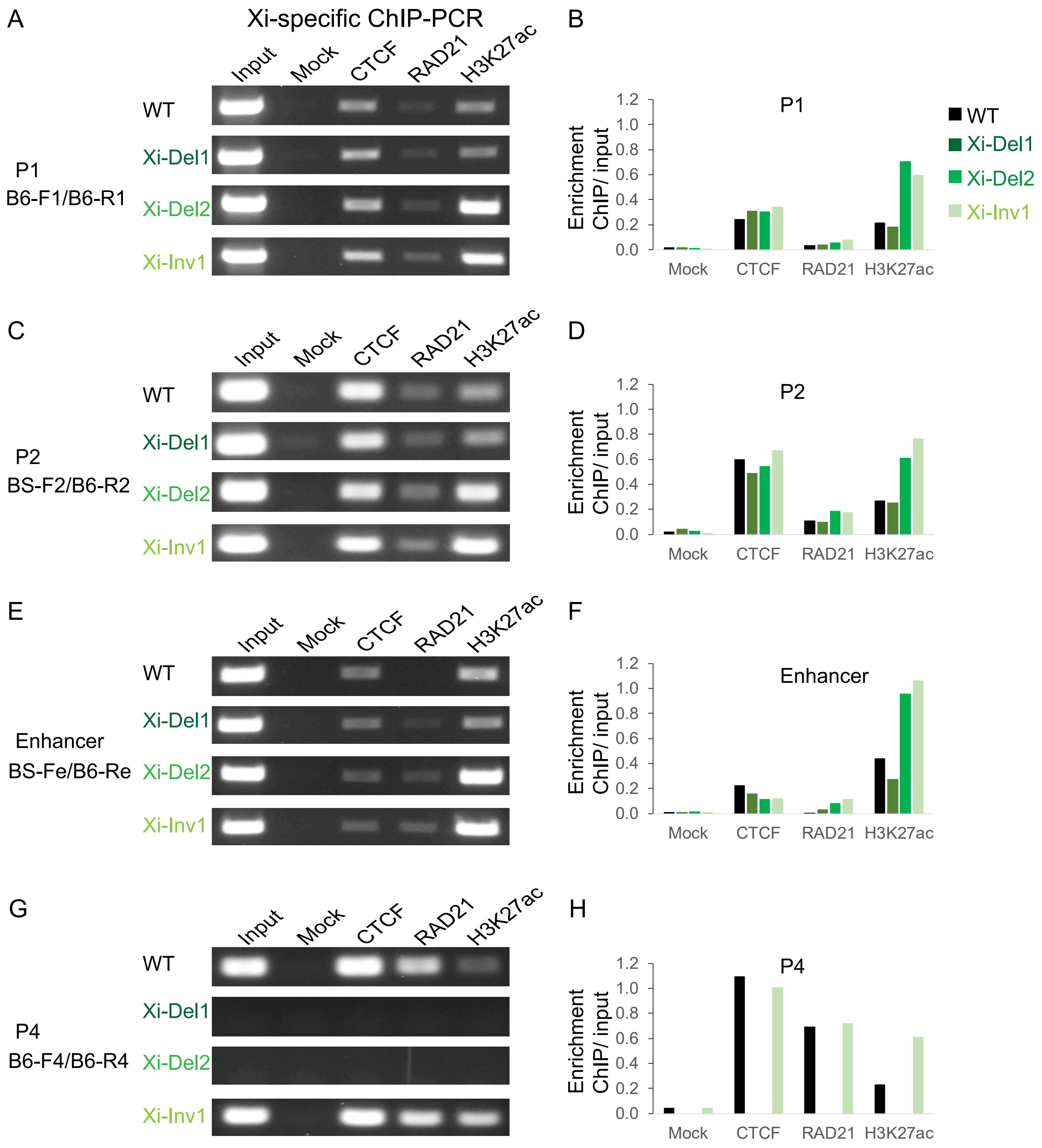
A
Xi-specific ChIP-PCR
Input
Mock
CTCF
RAD21
H3K27ac
WT
Xi-Del1
Xi-Del2
Xi-Inv1
P1
B6-F1/B6-R1
B
P1
Enrichment ChIP/ input
1.2
1.0
0.8
0.6
0.4
0.2
0.0
Mock
CTCF
RAD21
H3K27ac
WT
Xi-Del1
Xi-Del2
Xi-Inv1
C
P2
BS-F2/B6-R2
D
P2
E
Enhancer
BS-Fe/B6-Re
F
Enhancer
G
P4
B6-F4/B6-R4
H
P4

Fig. S12

Fig. S11

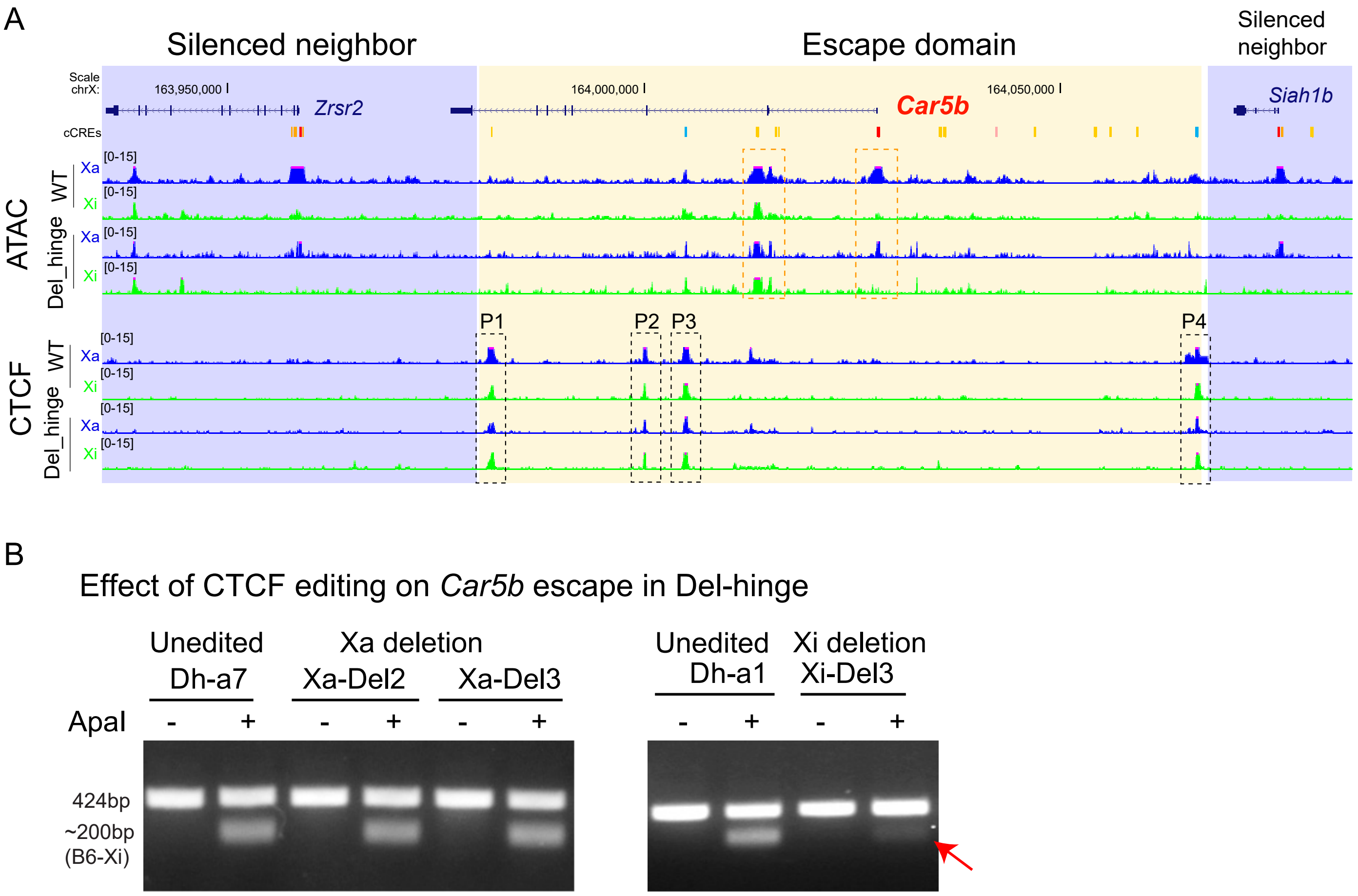

Fig. S12

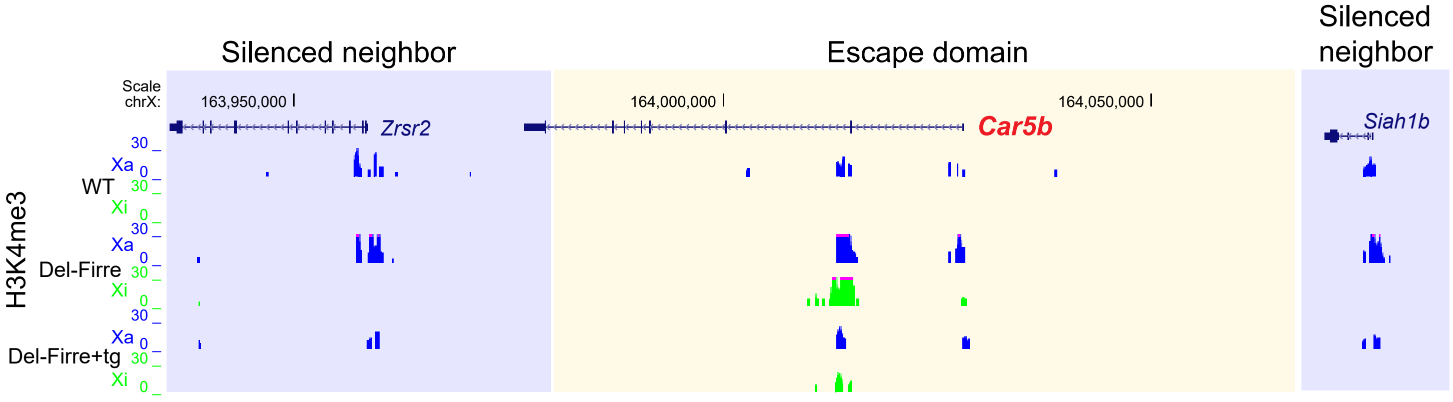
Silenced neighbor
Escape domain
Silenced neighbor
Scale
chrX:
163,950,000
164,000,000
164,050,000
Zrsr2
Car5b
Siah1b
H3K4me3
WT
Del-Firre
Del-Firre+tg
Xa
Xi
30
0

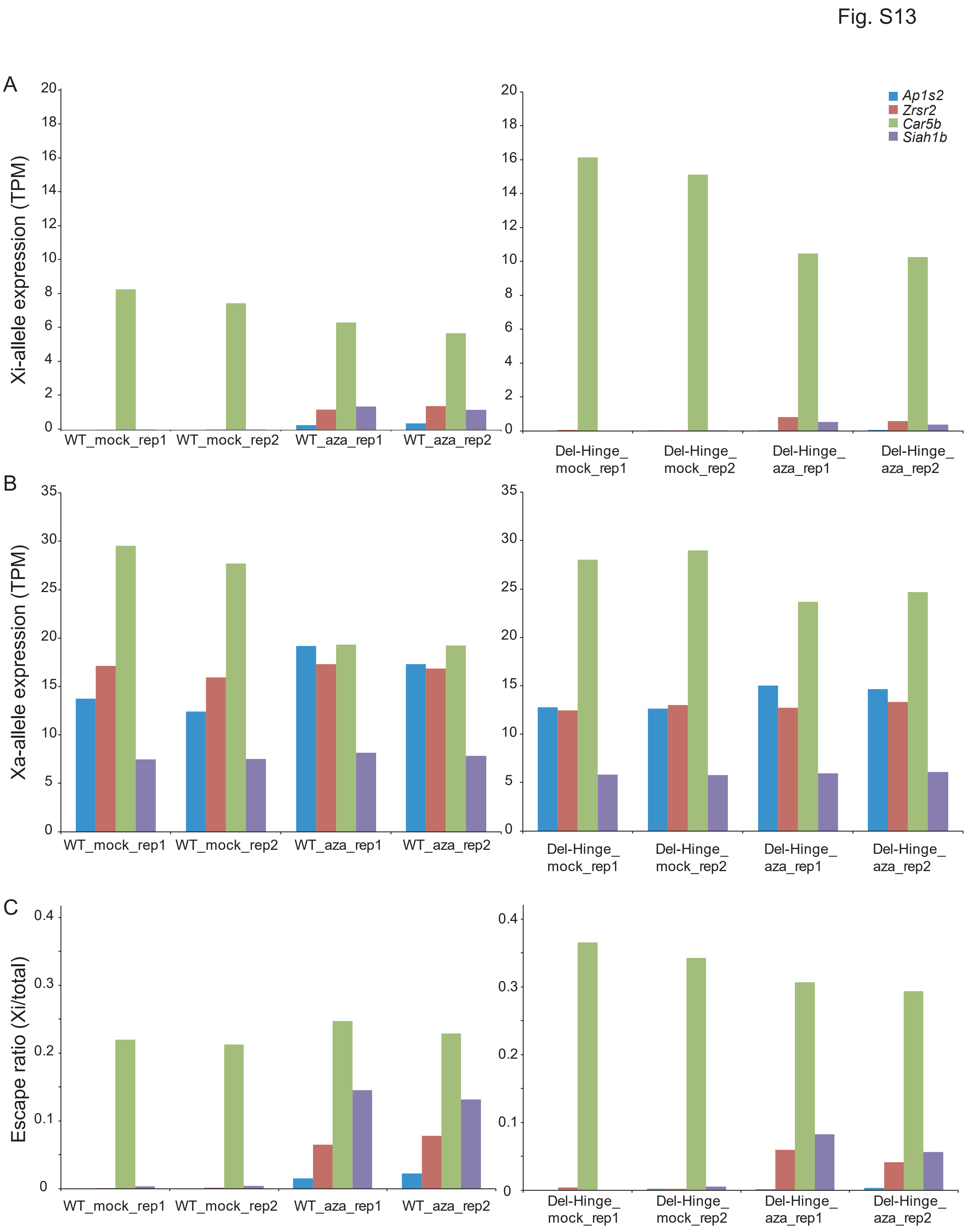
Fig. S13
A
B
C
Xi-allele expression (TPM)
Xa-allele expression (TPM)
Escape ratio (Xi/total)
Ap1s2
Zrsr2
Car5b
Siah1b
WT_mock_rep1
WT_mock_rep2
WT_aza_rep1
WT_aza_rep2
Del-Hinge_ mock_rep1
Del-Hinge_ mock_rep2
Del-Hinge_ aza_rep1
Del-Hinge_ aza_rep2
