# Supplementary material for: CTCF-mediated insulation and chromatin environment modulate Car5b escape from X inactivation
Source: BMC Biol. 2025 Mar 3;23:68. doi: 10.1186/s12915-025-02137-7 (PMC11874400; doi:10.1186/s12915-025-02137-7)

Uncropped DNA gels

Figure 5B

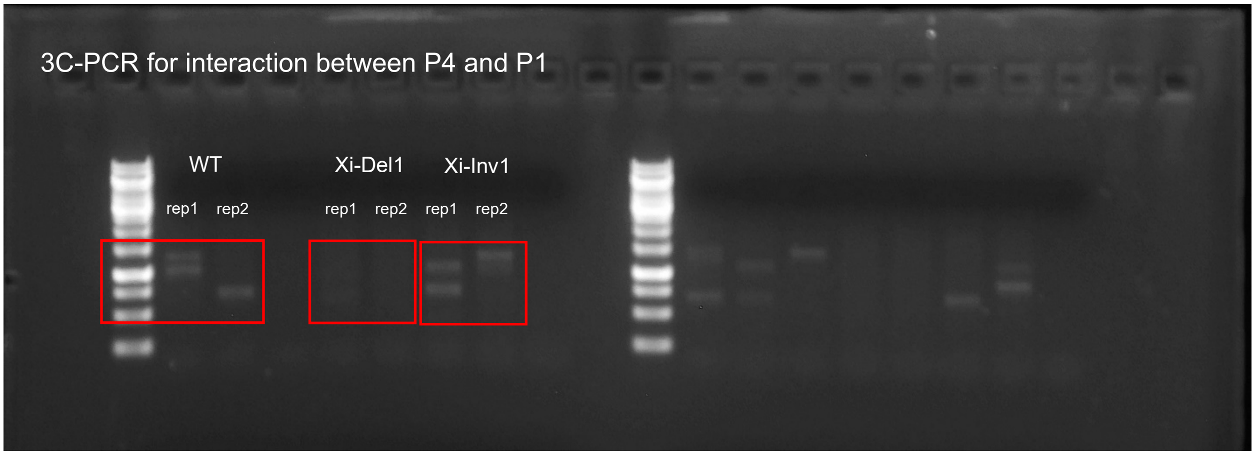

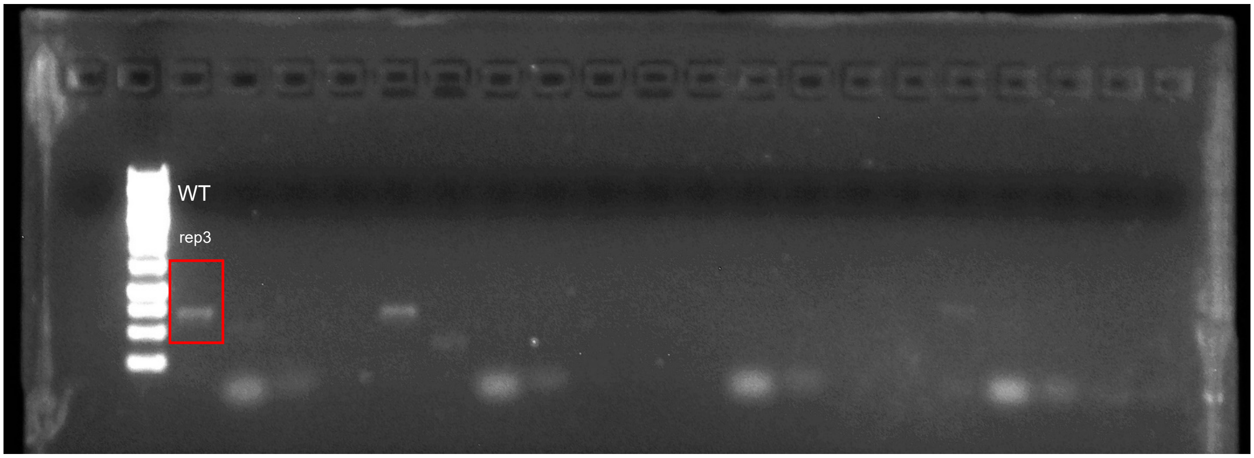

Figure 5C

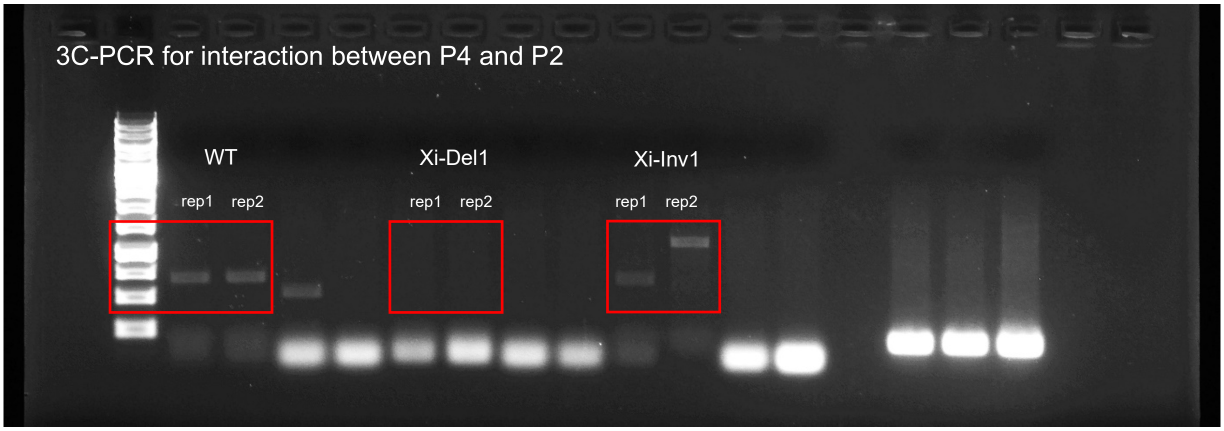

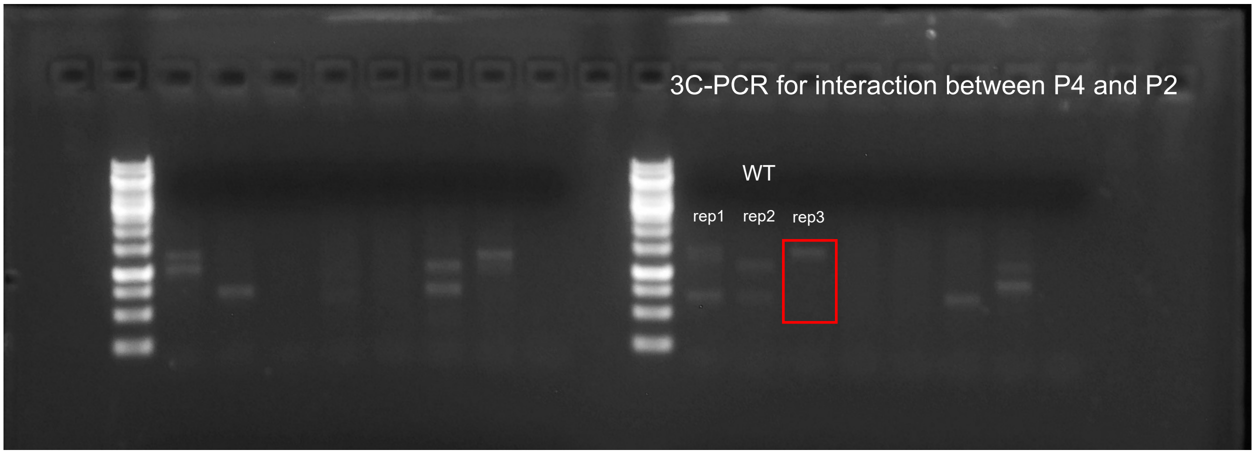

gDNA PCR for Figure 5B, C

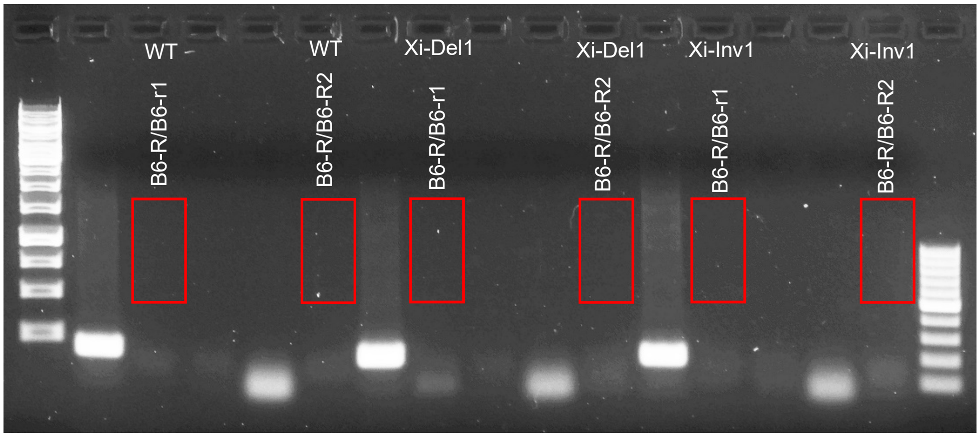

Figure S4C

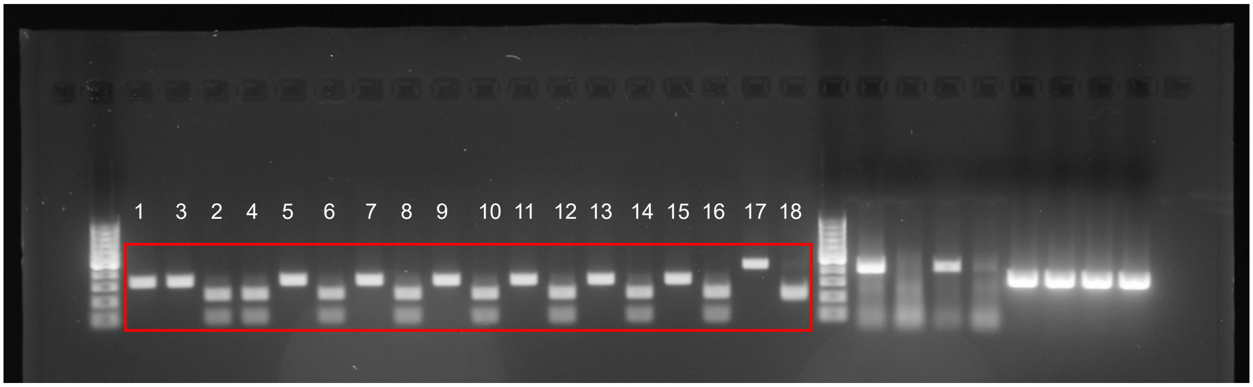

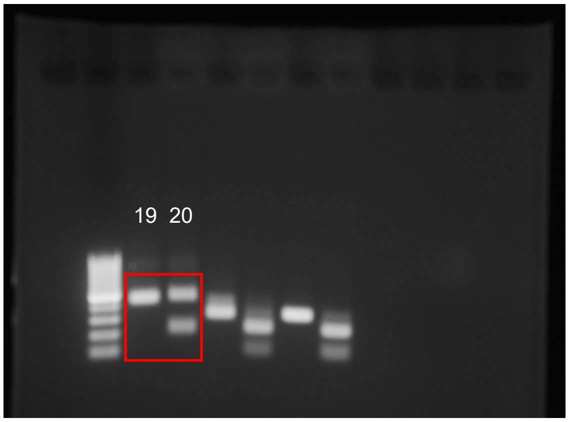

Lanes are numbered based on the order in Figure S4C. Lanes 1-16 are PCR products with cDNA from mouse tissues and MEFs followed by mock or B6-specific ApaI digestion. Lanes 17-20 are PCR products from genomic DNA followed by mock or B6-specific ApaI digestion. Note that lane 2 was switched with lane 3 in Fig S4C due to a sample loading mistake.

Figure S5B mid-panel

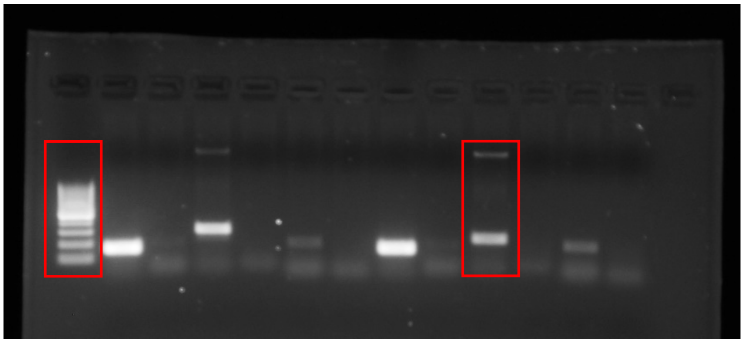

Figure S5C

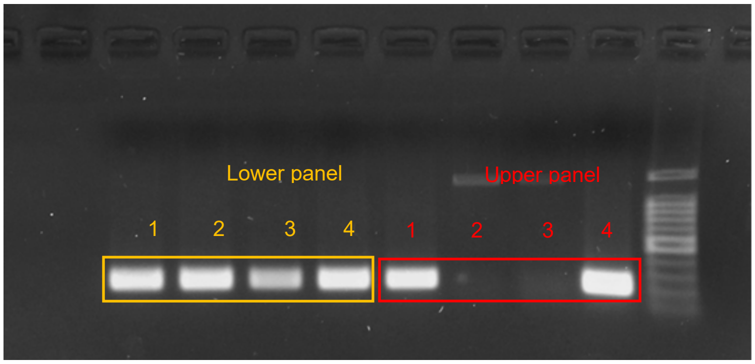

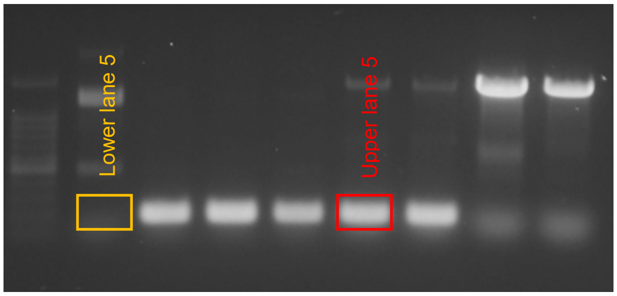

Figure S6A

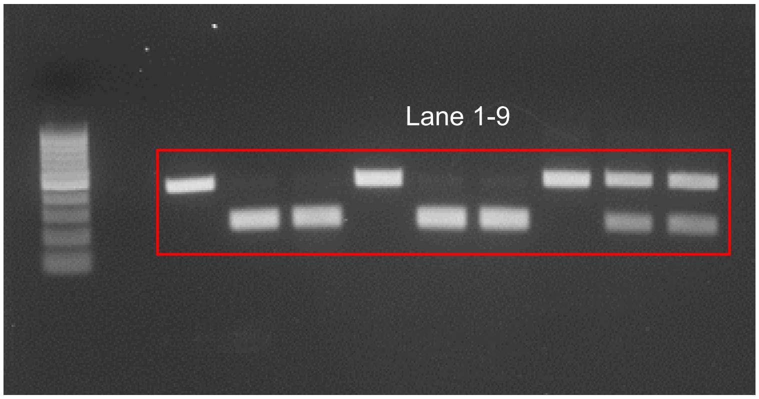

Figure S6B

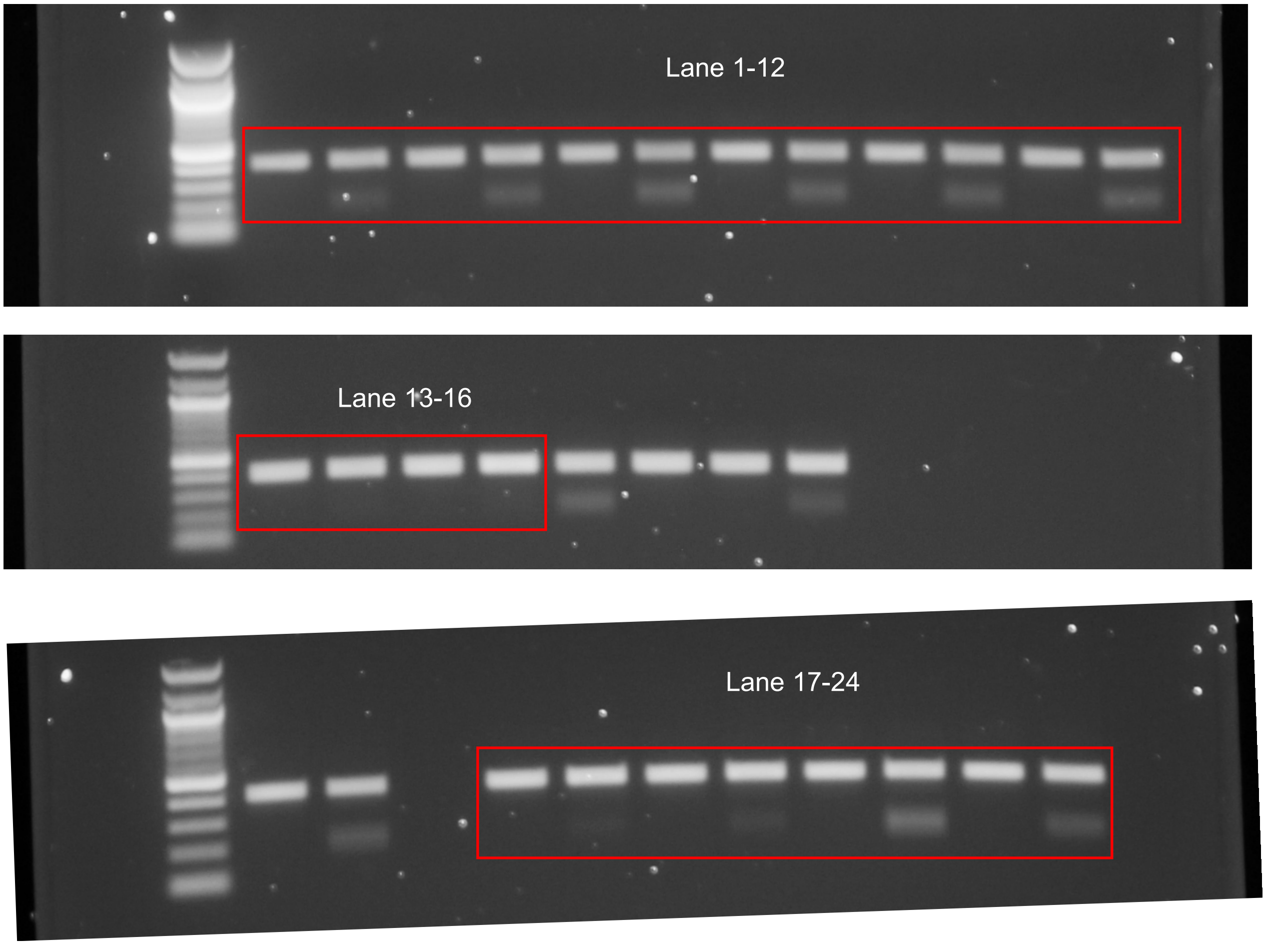

Figure S6D

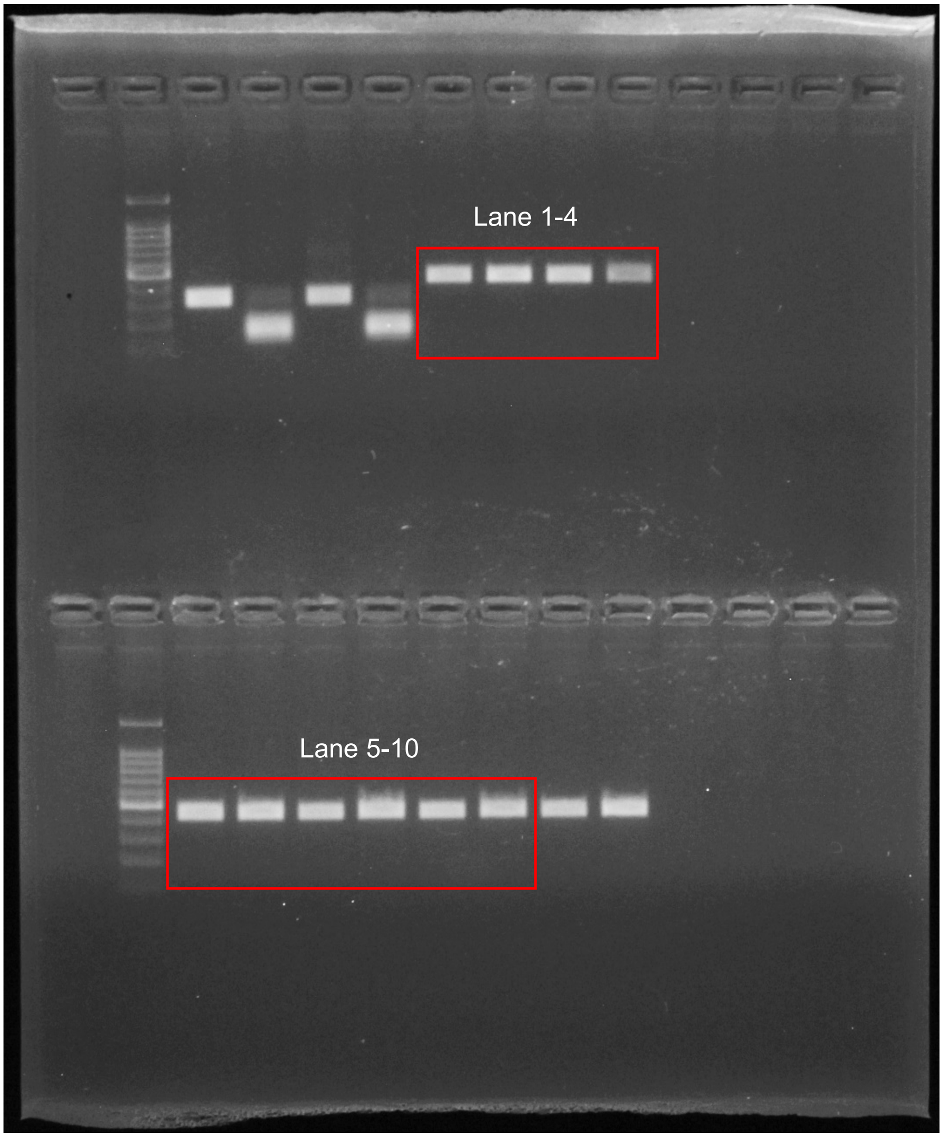

Figure S8A

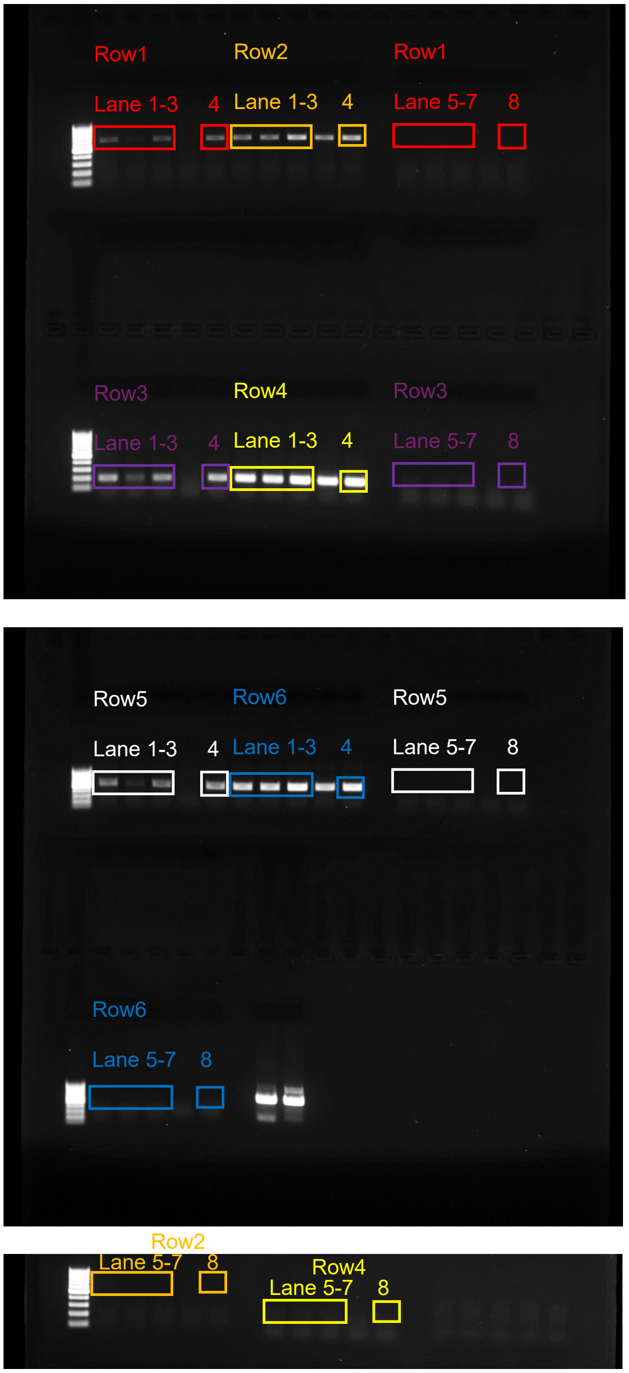

Row1
Row2
Row1
Lane 1-3
4
Lane 1-3
4
Lane 5-7
8
Row3
Row4
Row3
Lane 1-3
4
Lane 1-3
4
Lane 5-7
8
Row5
Row6
Row5
Lane 1-3
4
Lane 1-3
4
Lane 5-7
8
Row6
Lane 5-7
8
Row2
Lane 5-7
8
Row4
Lane 5-7
8

Figure S10

Fig. S10A for P1

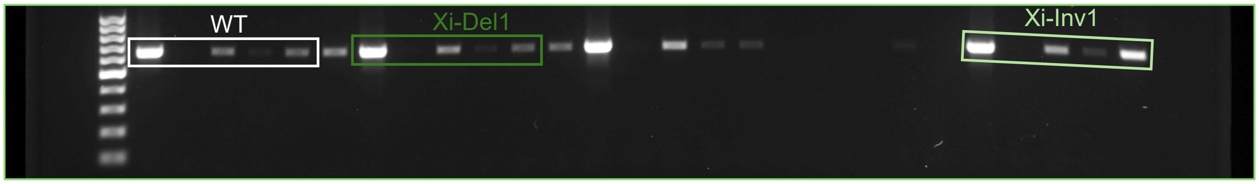

Fig. S10C for P2

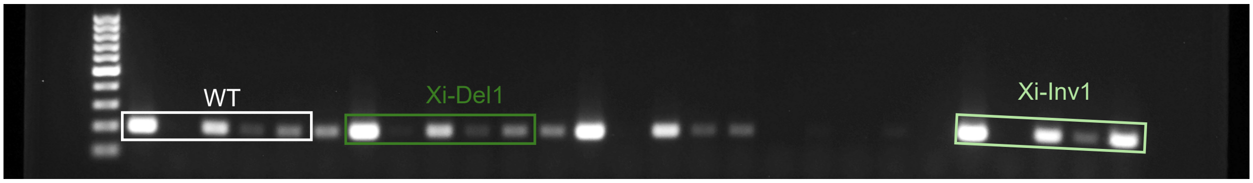

Fig. S10E for enhancer

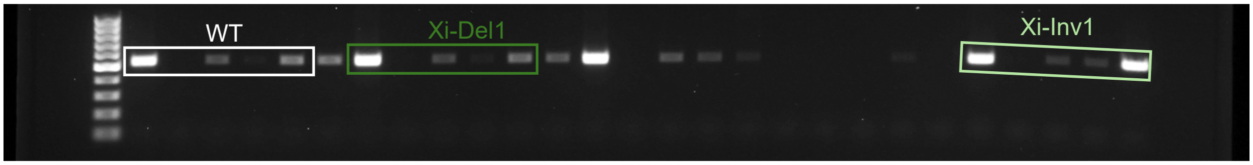

Fig. S10G

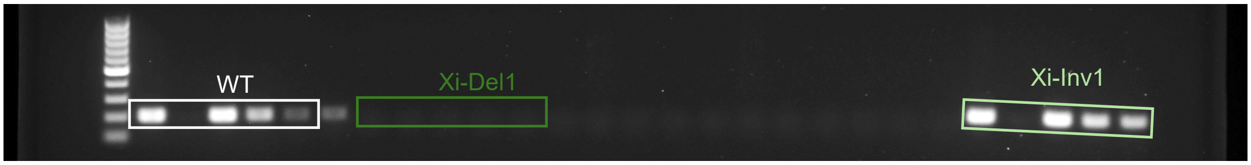

ChIP-PCR for Xi-Del2 at tested regions

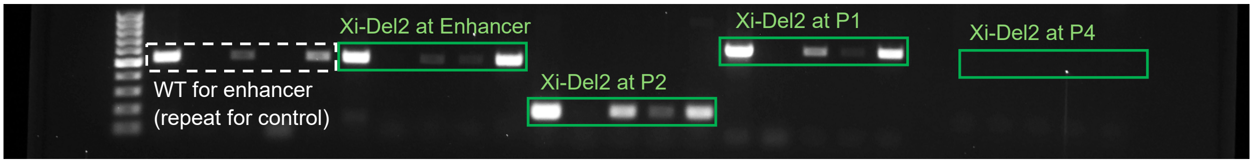

Figure S11B

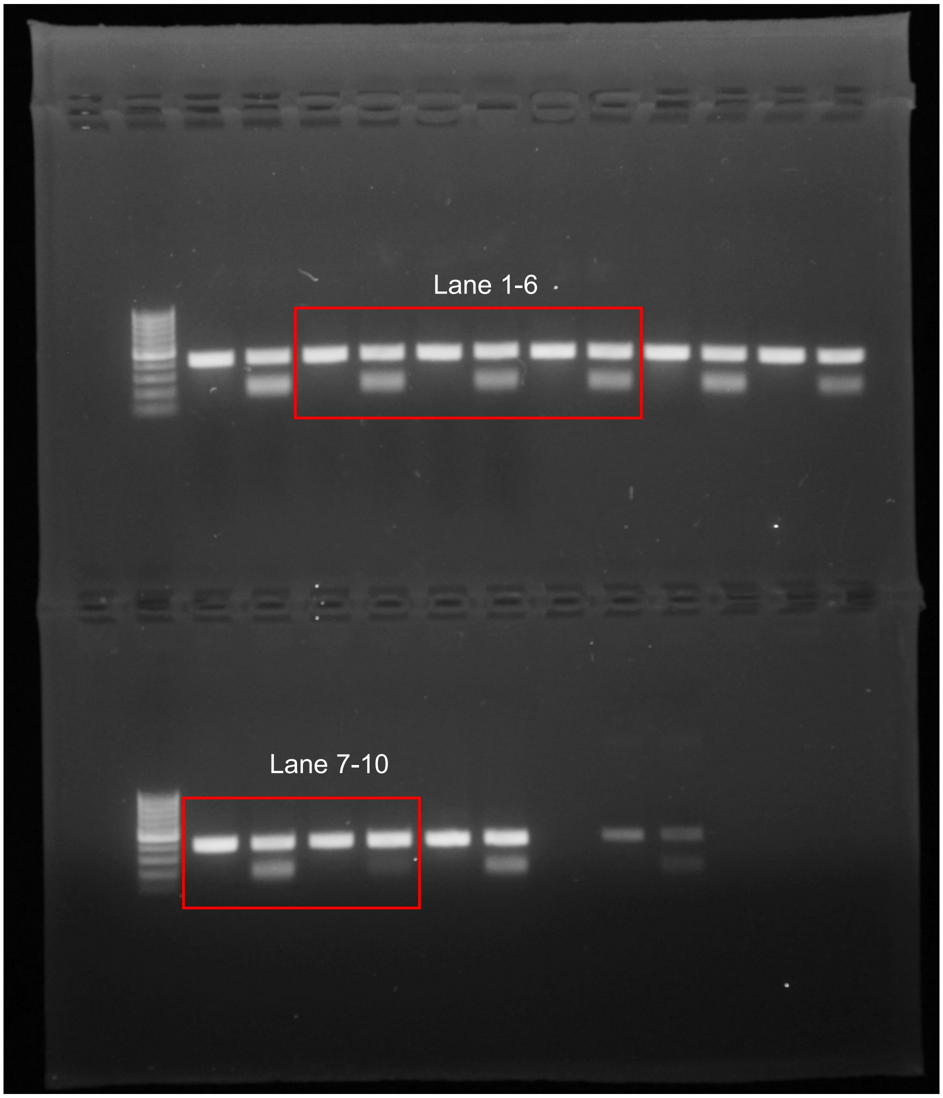

Supplement: Supplementary file 3 — Additional file 3: Uncropped DNA gel images [file 12915_2025_2137_MOESM3_ESM.pdf]
